# Optimized enrichment of circulating extracellular vesicles from whole blood samples using PROSPR

**DOI:** 10.20517/evcna.2026.18

**Published:** 2026-07-07

**Authors:** Jose Antonio Sánchez Milán, María Mulet, Maria Font-Alberich, Magdalena Torres, Yormaris Castillo, Silvia Pico, Oriol Yuguero, Gerard Piñol-Ripoll, Filip Bellon, Montserrat Gea-Sánchez, Siu Kwan Sze, Raj N. Kalaria, Xavier Gallart-Palau, Aida Serra

**Affiliations:** ^1^Biomedical Research Institute of Lleida Dr. Pifarré Foundation (IRBLleida), +Pec Proteomics Research Group (+PPRG), Neuroscience Area, Lleida 25198, Spain.; ^2^Department of Medical Basic Sciences, University of Lleida (UdL), Lleida 25198, Spain.; ^3^Laboratory Medicine Department, University Hospital Arnau de Vilanova, Lleida 25198, Spain.; ^4^ERLab Research Group, Biomedical Research Institute of Lleida Dr. Pifarré Foundation (IRBLleida), Lleida 25198, Spain.; ^5^Department of Medicine, Faculty of Medicine, University of Lleida (UdL), Lleida 25198, Spain.; ^6^Clinical Neuroscience Research, Unitat Trastorns Cognitius, Biomedical Research Institute of Lleida Dr. Pifarré Foundation (IRBLleida), Santa Maria University Hospital, Lleida 25198, Spain.; ^7^Healthcare Research Group (GRECS), Biomedical Research Institute of Lleida Dr. Pifarré Foundation (IRBLleida), Lleida 25198, Spain.; ^8^Grup d’Estudis Salut, Societat, Educació i Cultura de les Cures (GESEC), Departament d’Infermeria i Fisiotèrapia, Universitat de Lleida, Lleida 25198, Spain.; ^9^CIBER Fragilidad y Envejecimiento Saludable (CIBERFES), Instituto de Salud Carlos III, Lleida 25198, Spain.; ^10^Department of Health Sciences, Faculty of Applied Health Sciences, Brock University, St. Catharines L2S 3A1, Canada.; ^11^Translational and Clinical Research Institute, Campus for Ageing and Vitality, Newcastle University, Newcastle upon Tyne NE4 6BE, UK.; ^12^Biomedical Research Institute of Lleida Dr. Pifarré Foundation (IRBLleida), +Pec Proteomics Research Group (+PPRG), Neuroscience Area, University Hospital Arnau de Vilanova (HUAV), Lleida 25198, Spain.

**Keywords:** Extracellular vesicle enrichment, PROSPR workflow, whole-blood biobanking, untargeted lipidomics, high-depth proteomics, multi-omics integration, circulating biomarkers

## Abstract

**Aim:** Whole blood constitutes a robust and readily available resource for the identification and characterization of systemically circulating substances. Despite the relevant number of blood specimens archived in biobanks globally, their utilization in extracellular vesicle (EV) research remains considerably underexploited. In this study, we present an optimized PRotein Organic Solvent PRecipitation (PROSPR) workflow, specifically designed for the rapid and standardized enrichment of EVs directly from untreated blood samples.

**Methods:** PROSPR workflow was applied for EV enrichment directly from untreated blood. Comprehensive biophysical characterization was performed using dedicated flow cytometry, tunable resistive pulse sensing, and transmission electron microscopy. In parallel, discovery-driven lipidomics and high-depth proteomics were applied to assess the suitability of PROSPR-derived EVs for advanced systems biology and multi-omics integration.

**Results: **PROSPR consistently yielded highly purified EV preparations that preserved canonical vesicular morphology, structural integrity, and stable ζ-potentials, indicative of intact membrane characteristics. Multi-omics analyses revealed distinct and coordinated lipid-protein signatures uniquely enriched in EV populations derived from whole blood. These signatures predominantly reflected platelet- and mitochondria-associated cargo that was not detectable in corresponding plasma-derived EV fractions, highlighting key molecular features missed by plasma-based EV profiling approaches.

**Conclusion:** Consequently, the optimized PROSPR workflow presented here offers a robust and accessible methodological strategy to fully utilize dormant, archived blood resources within global and national biobanks, thereby enabling comprehensive mechanistic insights and facilitating the discovery of novel diagnostic and prognostic circulating biomarkers.

## INTRODUCTION

Blood is a vital systemic fluid that plays a key role in reflecting the physiological state of an organism^[1]^. The molecular richness of blood provides valuable insights into homeostatic conditions and altered states. Its molecular complexity stems from its diverse range of components, including red and white blood cells, platelets, proteins, metabolites, lipids, nucleic acids, and extracellular vesicles (EVs)^[1]^.

EVs, including exosomes and microvesicles, are small membrane-bound particles that play critical roles in intercellular communication^[2,3]^. Abundant in blood, EVs contain a diverse cargo of biomolecules, such as proteins, nucleic acids, and lipids^[2]^. This unique composition makes blood EVs valuable biomarkers. EVs facilitate the transfer of genetic material and proteins between cells, thereby influencing physiological processes^[2]^. Changes in the EV composition or concentration can serve as indicators of disease progression, treatment response, and overall health status^[3]^. For example, alterations in tumor-derived EVs with circulating capacity in the blood may provide insights into cancer development and metastasis, while EVs from immune cells may reflect inflammatory conditions or autoimmune disorders^[4]^. Consequently, the study of circulating EVs offers significant potential for understanding complex biological processes and disease mechanisms.

Advanced analytical methods, such as mass spectrometry and multiplexed immunoassays, enable the simultaneous detection of multiple analytes and offer comprehensive molecular profiles crucial for disease biomarker identification, drug metabolism studies, and physiological monitoring^[5-7]^. Despite the broad applicability of these technologies, blood analysis often prioritizes the plasma fraction, owing to its molecular richness and intrinsic characteristics^[8,9]^. Plasma, the liquid component of the blood, contains proteins, metabolites, EVs, and other biomarkers^[8]^. Its molecular diversity provides a comprehensive molecular profile, whereas the absence of cellular components enhances stability and reduces interference in molecular assays, resulting in more accurate results^[8]^. Nevertheless, while plasma offers significant advantages for molecular analyses, obtaining it requires several processing steps that may inadvertently remove important molecular features present in the whole blood^[10]^. Specifically, plasma separation may also affect the yield and characteristics of the obtained EVs. By discarding a portion of these components during plasma preparation, valuable biomarkers that may be crucial for understanding disease mechanisms and developing therapeutic strategies are lost^[10]^. Therefore, while plasma is more stable and reduces interference in assays, whole blood may offer an alternative molecular profile for specific EVs subsets, particularly when focusing on molecular profiling of EVs directly from whole blood.

In clinical settings, it is imperative to customize strategies based on the specific analysis to be conducted, particularly when utilizing blood samples. However, owing to technical limitations in clinical laboratories^[11,12]^, substantial quantities of blood are frequently collected. These constraints, such as restricted processing time or insufficient equipment^[11]^, often result in the storage of whole blood instead of plasma, which is generally preferred for molecular analyses. Consequently, such whole blood samples represent a valuable source of biomarkers, they may be underutilized or unsuitable for numerous contemporary molecular profiling methodologies.

Some years ago, members of our research team developed a method known as PRotein Organic Solvent PRecipitation (PROSPR), which leverages the hydrophobic properties of EVs in an environment induced by organic solvents^[13,14]^. PROSPR functions by creating a highly hydrophobic environment that facilitates the isolation of EVs based on their inherent characteristics, thus rendering it an efficient and reliable technique^[13]^. In contrast to other enrichment methods, PROSPR offers a cost-effective and straightforward protocol that requires minimally specialized equipment. If successfully adapted for use with blood as the starting material, PROSPR could provide a practical solution for the molecular profiling of EVs, particularly when plasma has not been extracted due to technical constraints, as previously mentioned. Furthermore, PROSPR may offer an advantage in clinical settings when the analysis of specific subsets of EVs is desired, enabling the circumvention of plasma separation through a rapid and readily implemented protocol to obtain EVs circulating in whole blood. However, it remains uncertain whether PROSPR can effectively enrich EVs from whole blood and how the obtained EVs would facilitate omics analyses. Additionally, it is unclear how the resulting subsets would differ from or resemble those extracted from plasma. To address these crucial methodological and translational aspects, this study conducted a thorough and comprehensive investigation of whole blood PROSPR-enriched EVs.

## METHODS

### Reagents

All chemicals were procured from Sigma-Aldrich (St. Louis, MO, USA), unless otherwise specified. High-performance liquid chromatography (HPLC)-grade water and acetonitrile (ACN) were supplied by Thermo Fisher Scientific (USA). Sequencing-grade modified trypsin was obtained from Promega (Madison, WI, USA).

### Sample collection and preprocessing

Whole blood (blood) samples were obtained from elderly donors without documented diagnosed pathologies (*n* = 5) and stored at -80 °C, as provided by the Newcastle Tissue Resource (NBTR), Newcastle upon Tyne, United Kingdom. An independent validation cohort of blood and ethylenediaminetetraacetic acid (EDTA)-plasma samples was also collected at the University Hospital Arnau de Vilanova (HUAV), Lleida, Spain (blood, *n* = 5; plasma, *n* = 10) and supplied by the HUAV Biobank.

For blood samples, the samples were maintained at -80 °C until analysis. For plasma samples, immediately following venepuncture, EDTA was added to each tube to achieve a final concentration of 20 IU/mL. The samples were then centrifuged at 4,200 × *g* for 10 min at room temperature. Plasma-depleted cellular fractions (cellular fraction) corresponded to the remaining blood cellular component obtained after plasma separation by centrifugation. Plasma and cellular fractions were aliquoted, snap-frozen and stored at -80 °C until required for use. Comparative analyses were performed using independent biological samples.

All experimental procedures adhered to the Declaration of Helsinki and received approval from the HUAV Ethics Committee (Ref# CEIC-3089). The experimental work was conducted in strict compliance with relevant European, national, and institutional biosafety and data protection regulations. Informed consent was obtained from all participants or their legally authorized representatives at the respective biobanks involved.

### Enrichment of EVs

EVs derived from blood and plasma were enriched using the PROSPR protocol, as previously documented^[13,15]^. In brief, freshly thawed aliquots of plasma (200 µL) or blood (50-200 µL) were transferred to pre-chilled glass tubes maintained on ice and immediately combined with four volumes of acetone pre-equilibrated to -20 °C. The samples were vortexed for 5 s to ensure complete dispersion and subsequently centrifuged at 6,000 × *g* for 1 min at 4 °C. The resultant proteinaceous pellet was discarded, and the supernatant, containing the hydrophobic, EV-enriched fraction, was transferred to a clean 1.5 mL tube and concentrated to near dryness in a refrigerated vacuum concentrator (Eppendorf, Hamburg, Germany). The concentrated EV preparations were sealed and stored at -80 °C until further analysis.

### Dedicated flow cytometry

EVs were analyzed by dedicated flow cytometry (dFC) using a CytoFLEX SRT sorter (Beckman Coulter, Brea, USA) equipped with 405, 488, 561, and 638 nm lasers. Side scatter (SSC) data were collected from the 405 nm (violet) laser to enhance sensitivity for particles smaller than 200 nm, and red fluorescence was recorded through a 660/10 nm band-pass filter (R1). Instrument performance was verified at the beginning of each session: volumetric flow rate, fluorescence gain, and SSC alignment were adjusted using Daily QC Calibration beads (Beckman Coulter, Brea, USA), and the SSC detection threshold was established with Megamix Plus SSC beads (Stago Diagnostica, Asnières-sur-Seine, France), which cover the 100-900 nm diameter range.

Data acquisition was initiated based on red fluorescence, with a threshold set at 1,500 arbitrary units, to mitigate electronic noise. A control sample containing only buffer (0.1 µm-filtered dPBS) was recorded for a duration of 5 min at a rate of less than 2 events per second, using identical settings to establish background levels and define gate boundaries. EV samples were processed for 5 min at a rate of 300-500 events per second in low-flow mode (10 µL per minute) to minimize coincident detection. The data were exported as FCS 3.0 files and subsequently analyzed using FlowJo version 10.8 (BD Biosciences, Franklin Lakes, USA).

A four-step gating hierarchy was employed to ensure the retention of only single, EV-sized events. (i) Time gate: A plot of Time versus R1 was utilized to identify a continuous region devoid of flow spikes; (ii) Singlet gate: Pulse width (R1-Width) was plotted against pulse height (R1-Height); events located on the main diagonal were retained, while wider “swarm” events were excluded; (iii) EV gate: Singlets were displayed as violet SSC versus R1; the lower-left boundary was established such that less than 0.1% of dPBS events were included within the gate, and the 160 nm Megamix bead population was positioned just above both thresholds to define the lower size limit; (iv) Verification: Events within the EV gate were back-gated onto violet SSC versus green fluorescence (525/40 nm) to confirm the absence of bead carry-over or high-fluorescence artifacts. This strategy was uniformly applied to all samples.

### Ultrastructural characterization

Aliquots of 5 µL from three biological replicates per condition (blood and plasma) were filtered using a 300 kDa molecular weight cut-off (MWCO) filter (Amicon Ultra, Billerica, USA). These fractions were then applied to 300-mesh copper grids coated with Formvar and carbon film (Electron Microscopy Sciences, Hatfield, USA) and allowed to adsorb for 20 min at room temperature. The grids were subsequently rinsed twice with drops of HPLC-grade water (Fisher Chemical, Loughborough, UK), fixed for 5 min in 1% glutaraldehyde prepared in phosphate-buffered saline (Electron Microscopy Sciences, Hatfield, USA), and washed again with water. Negative staining was conducted using 1% (w/v) uranyl oxalate, pH 7.0 (Agar Scientific, Stansted, UK) for 5 min. Excess stain was removed with filter paper, and the grids were immediately embedded in a 2% methyl-cellulose/0.4% uranyl acetate solution (Sigma-Aldrich, St. Louis, USA) for 10 min, followed by air-drying on filter paper at room temperature.

Transmission electron microscopy (TEM) micrographs were acquired using a JEM-1010 transmission electron microscope (JEOL, Tokyo, Japan) operating at 80 kV and equipped with a digital Gatan Orius SC1000 charge-coupled device (CCD) camera (Gatan, Pleasanton, USA). The images were calibrated to the microscope scale bar, and contrast was enhanced using a standardized adjustment protocol. Vesicle size and morphology were analyzed using ImageJ v1.53 (National Institutes of Health, Bethesda, USA). To ensure a representative ultrastructural assessment, at least ten fields per grid and three independent grids per sample were examined.

### Tunable resistive pulse sensing

Tunable resistive pulse sensing (TRPS) was employed for the comprehensive physicochemical characterization of EVs using an Exoid instrument (Izon Science, Christchurch, New Zealand). Calibration of the system was conducted immediately prior to each run utilizing CPC200 polystyrene standards (mean diameter 200 nm; Izon Science, Christchurch, New Zealand). EV suspensions were processed using a 300 kDa MWCO filter (Amicon Ultra, Billerica, USA) and subsequently diluted in 0.1 µm filtered phosphate-buffered saline (PBS; Sigma-Aldrich, St. Louis, USA) to achieve an event rate of 100-400 particles per minute. Measurements were conducted with an NP200 polyurethane nanopore (Izon Science, Christchurch, New Zealand), which offers optimal resolution for particles ranging from 85 to 500 nm. The parameters of stretch, voltage, and hydraulic pressure were maintained constant during both calibration and subsequent size and concentration determinations; ζ-potential was evaluated under the same stretch setting while voltage and pressure were varied during calibration and maintained during ζ-potential analysis. Data acquisition was continued until a minimum of 250 valid particle blockades were recorded per sample. All traces were collected and analyzed using Control Suite software v3.4 (Izon Science, Christchurch, New Zealand).

### Bicinchoninic-acid assay

The total protein content in the EV preparations was quantified using the bicinchoninic acid (BCA) assay. The working reagent was freshly prepared by mixing the BCA solution (B9643; Supelco, Darmstadt, Germany) with a 4% (w/v) copper(II) sulfate solution (C2284; Sigma-Aldrich, St. Louis, USA) in a 50:1 ratio, in accordance with the manufacturer’s instructions. Aliquots of 25 µL of either the sample or bovine serum albumin standards (0-2 mg/mL) were dispensed into a clear, flat-bottom 96-well plate (Corning, New York, USA) and combined with 200 µL of the working reagent. Following incubation at 37 °C for 30 min, absorbance was measured at 562 nm using a Synergy HTX microplate reader (BioTek Instruments, Winooski, USA). Protein concentrations were determined by linear regression against the bovine serum albumin (BSA) standard curve, with all samples analyzed in technical triplicate.

### Lipid extraction and untargeted lipidomics

For the extraction of lipids, 5 µL of PROSPR-enriched EVs from blood and plasma were combined with 5 µL of Milli-Q water and 20 µL of ice-cold methanol (Optima LC-MS grade, Thermo Fisher Scientific, Waltham, USA). The samples were subjected to vigorous vortexing for 2 min, followed by the addition of 250 µL of methyl tert-butyl ether (MTBE) (Sigma-Aldrich, St. Louis, USA). The mixture underwent ultrasound-assisted extraction by immersion in a water bath sonicator (ATU Ultrasonidos, Valencia, Spain) operating at 40 kHz and 100 W for 30 min at 10 °C. After sonication, 25 µL of Milli-Q water was added to facilitate phase separation. The samples were then centrifuged at 1,400 × *g* for 10 min at 10 °C. The resultant upper organic phase, containing the EV-derived lipids, was meticulously collected and transferred to glass autosampler vials for further analysis. Stock solutions were formulated by dissolving lipid standards in MTBE at a concentration of 1 mg/mL, and subsequently, working solutions were diluted to 2.5 µg/mL in MTBE.

PROSPR-EV lipid extracts were subjected to Liquid Chromatography-Electrospray Ionization Quadrupole Time-of-Flight Mass Spectrometry (LC-ESI-Q-TOF) analysis using an Agilent 1290 Infinity II Ultra-High-Performance Liquid Chromatography (UHPLC) system, which was coupled to a 6545 Q-TOF mass spectrometer equipped with a dual ESI source (Agilent Technologies, Santa Clara, USA). The autosampler was maintained at a temperature of 4 °C, and 10 µL of each extract was injected onto a Waters ACQUITY HSS T3 column (100 mm × 2.1 mm, 1.8 µm; Waters, Milford, USA) maintained at 55 °C. Chromatographic separation was conducted at a flow rate of 0.40 mL/min using solvent A (10 mM ammonium acetate in ACN/water, 40:60 v/v) and solvent B (10 mM ammonium acetate in ACN/isopropanol, 10:90 v/v). A linear gradient was employed, transitioning from 40% to 100% solvent B over 10 min, followed by a 2-min hold at 100% B, and a 3-min re-equilibration at 60% B, resulting in a total run time of 15 min. The Q-TOF mass spectrometer acquired full-scan spectra (m/z 100-3000, 2 GHz, 1 scan/s) in separate positive- and negative-ion modes, utilizing nitrogen as the nebulizer gas (5 L/min, 350 °C) with a capillary voltage of 3.5 kV. Continuous mass-axis calibration was achieved through dual-spray infusion of reference ions (m/z 121.050873 and 922.009798 in positive mode; m/z 119.036320 and 966.000725 in negative mode).

### Proteomes in-solution processing

PROSPR-enriched EV preparations from blood and plasma containing approximately 20 µg of total protein, were lysed in a solution containing 8 M urea and 100 mM Tris-HCl at pH 8.5. These preparations were subsequently loaded onto 30 kDa MWCO centrifugal filter units (Amicon Ultra, Darmstadt, Germany). Following centrifugation at 14,000 × *g* for 15 min, the retained proteins were reduced on the filter with 10 mM dithiothreitol in the lysis buffer for 30 min at 37 °C. This was followed by alkylation with 20 mM iodoacetamide for 20 min in the dark at room temperature. The filters were then washed twice with the lysis buffer and three times with 50 mM ammonium bicarbonate to remove residual urea. Proteolysis was initiated by the addition of sequencing-grade trypsin (Promega, Madison, USA) at an enzyme-to-protein ratio of 1:50 (w/w) in 50 mM ammonium bicarbonate, with incubation overnight at 37 °C. The resulting peptides were recovered by centrifugation, rinsed from the filter with an additional 50 µL of ammonium bicarbonate, and acidified to 0.5% formic acid to terminate digestion. The peptide mixtures were desalted using 50 mg Sep-Pak C18 cartridges (Waters, Milford, USA), eluted with a solution of 70% acetonitrile/0.1% formic acid, and subsequently dried in a vacuum concentrator prior to liquid chromatography-tandem mass spectrometry (LC-MS/MS) analysis.

### Discovery-driven proteomics

Desalted PROSPR-enriched EV peptide mixtures from blood and plasma were reconstituted in 0.1% formic acid and introduced into an Evosep One nanoflow liquid chromatography system (Evosep, Odense, Denmark) operating at a flow rate of 300 nL/min, utilizing an 88-min stepped gradient on a 15 cm × 150 µm C18 analytical column (PepSep, Odense, Denmark) maintained at 40 °C. The Evosep system was interfaced online with a timsTOF Pro mass spectrometer (Bruker Daltonics, Billerica, USA) equipped with a trapped-ion mobility spectrometry (TIMS) device and operated in four-dimensional parallel-accumulation-serial-fragmentation (PASEF) mode. Survey scans were conducted over an m/z range of 100-1,700 with an ion-mobility window of 0.60-1.60 V·s/cm^2^, employing a 100 ms TIMS ramp and ten PASEF MS/MS frames per acquisition cycle (cycle time ≈ 1.1 s). Collision-induced dissociation energy was linearly adjusted as a function of ion mobility, and precursors were dynamically excluded for 0.4 min. External calibration was performed daily using the Bruker ESI-LC tuning mix to ensure mass accuracy of less than 1 ppm throughout the acquisition.

### Lipidomics data processing

In the processing of lipidomics data, raw LC-ESI-Q-TOF files derived from the characterization of PROSPR-enriched EVs from blood and plasma were analyzed using MassHunter Qualitative Analysis B.07.00 (Agilent Technologies, Barcelona, Spain). The Molecular Feature Extractor (MFE) algorithm was employed to group co-eluting adducts of identical molecular entities. Molecular features comprising two or more ions were exported to MassHunter Mass Profiler Professional for untargeted statistical analysis. Features were aligned with a retention time tolerance of 0.1% or 0.25 min and a mass tolerance of 30.0 ppm or 2.0 mDa; only those present in at least 70% of quality-control (QC) injections were retained. Signal drift was corrected using QC-based locally estimated scatterplot smoothing (LOESS) normalization, followed by the application of univariate analysis of variance (ANOVA) (*P* < 0.05) and multivariate partial least squares discriminant analysis (PLS-DA) to identify discriminatory lipids. Initial annotation of consistently detected features (*n* = 3 technical replicates) was conducted using CEU Mass Mediator v3.0 (mass accuracy < 10 ppm) and further refined against the Human Metabolome Database (HMDB) (< 30 ppm) and LIPID MAPS (< 20 ppm). Tentative identities were determined by searching for accurate matches to exact masses (tolerance ~0.01 Da), common lipid classes, and ion mode using LIPID MAPS, HMDB, and relevant scientific literature on lipid MS. In instances where no direct match was identified, the most probable class was suggested based on mass range and typical lipid profiles. Further statistical analysis of the lipidomic feature matrix was conducted using R (v4.5.0). Initially, molecular features were subjected to a two-sample *t*-test via the genefilter package (v1.90.0) to identify ions with significant differences between the experimental groups. Subsequently, the global structure of the dataset was examined through principal component analysis (PCA), implemented in FactoMineR (v2.11) and visualized with factoextra (v1.0.7). To evaluate sample-level clustering, hierarchical clustering combined with k-means partitioning was applied using the ComplexHeatmap package (v2.24.0), employing Euclidean distance as the similarity metric and Ward’s linkage for dendrogram construction.

### Proteomics data processing

In the analysis of proteomics data, raw timsTOF spectra from blood and plasma EVs were processed using PEAKS Studio X Pro v10.6 (Bioinformatics Solutions, Waterloo, Canada). Protein identification was conducted against the complete human reference proteome from UniProt (downloaded on 3 February 2023; comprising 140,065 entries), employing trypsin specificity and carbamidomethylation of cysteine as a fixed modification. The precursor and fragment mass tolerances were established at 10 ppm and 0.05 Da, respectively, with a requirement of at least one tryptic peptide per protein. Peptide-spectrum matches, peptides, and proteins were filtered to achieve a false-discovery rate of less than 1% through target-decoy analysis applied uniformly across all samples. Quantified proteins were cross-referenced with the top 100 EVs markers curated in ExoCarta and Vesiclepedia to determine EV provenance. Functional annotation and pathway enrichment were executed in R (v4.5.0) utilizing clusterProfiler (v4.6.2) and org.Hs.eg.db (v3.16.0), with the application of Benjamini-Hochberg correction, considering terms with an adjusted *P*-value of less than 0.05 as significant.

### Multi-omics integration

Proteomic and lipidomic feature matrices in comma-separated value (CSV) format were imported into MassHunter Mass Profiler Professional (Agilent Technologies, Santa Clara, USA). Prior to merging, each matrix underwent log transformation and Pareto scaling to balance variables of high and low abundance. Subsequently, a joint data frame was constructed through an inner join based on sample identifiers, and missing values (less than 5% per block) were imputed using the k-nearest neighbors method (*k* = 3). Unsupervised integration was examined via hierarchical clustering on the combined dataset, employing Euclidean distance and Ward’s linkage; the results were visualized as integrated heat maps to highlight concordant and discordant patterns across omics. To identify coordinated lipid-protein modules, Pearson correlation coefficients were computed for all cross-omic feature pairs, and significant associations were retained following Benjamini-Hochberg correction (*P* < 0.05). Correlated feature sets were subsequently analyzed using clusterProfiler for pathway enrichment, facilitating the functional interpretation of the multi-omics signatures.

### Data analysis

Quantitative data obtained from dFC, TEM, BCA, and TRPS assays were exported as CSV files and subsequently processed using custom macros in R (v4.5.0) and GraphPad Prism (v9.4.1). The normality of the data was evaluated using the Shapiro-Wilk test. In instances where normality was not met, group differences were analyzed using a non-parametric one-way ANOVA (Kruskal-Wallis); otherwise, a parametric one-way ANOVA followed by Tukey’s post-hoc correction was employed. Statistical significance was defined as *P* < 0.05, with adjustments made for multiple comparisons where necessary. Pearson correlation coefficients were calculated to assess pairwise variable associations, with |*r*| ≥ 0.8 considered indicative of a strong correlation.

To evaluate EV enrichment relative to proteins associated with non-vesicular extracellular proteins (nVEPs), an enrichment statistic was defined as follows:

**Figure:**
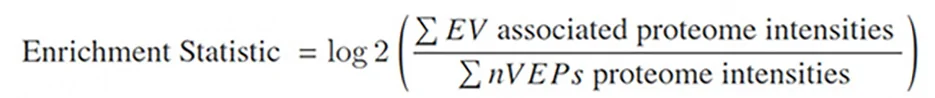


Protein-level intensities were first aggregated such that each protein was represented by a single value corresponding to the mean intensity across replicates, calculated in linear scale to ensure valid summation and ratio estimation. For benchmarking comparisons between PROSPR and differential ultracentrifugation followed by density gradient (dUC-DG) datasets, nVEP-associated proteins were categorized according to the classification approach applied in the reference dataset reported by Morales-Sanfrutos *et al.*^[16]^. This categorization includes major protein components of nVEPs, together with transmembrane, lipid-bound, and soluble proteins associated with intracellular compartments other than the plasma membrane or endosomes, which may contribute to coverage effects in mass spectrometry-based analyses. Comparative log2 enrichment ratios were subsequently calculated using the intensity values of proteins common to the datasets being compared.

Separately, an independent bootstrap-based internal validation analysis was performed exclusively on the PROSPR dataset using nVEP-associated protein categories defined according to the Minimal Information for Studies of Extracellular Vesicles (MISEV) framework for EV purity assessment^[17]^. In this analysis, nVEP-associated proteins included major components of non-EV co-isolated structures, such as lipoproteins, protein aggregates, and proteins associated with intracellular compartments other than the plasma membrane or endosomes. Bootstrap resampling (*n* = 1,000 iterations) was performed by sampling proteins with replacement from the complete dataset while preserving protein-level independence. For each iteration, the enrichment statistic was recalculated, and the 95% confidence interval was estimated using the percentile method (2.5th and 97.5th percentiles). Bootstrap analyses were implemented using base R functions together with the boot package (v1.3-31).

### Differential ultracentrifugation followed by density gradient

Differential ultracentrifugation (dUC) followed by density gradient (DG) EV proteomics datasets were retrieved from the publicly available study by Morales-Sanfrutos *et al.*^[16]^, which characterized serum-derived small EVs and non-vesicular components by DG-protein correlation profiling. In that study, crude small EV fractions were isolated from 4 mL human serum samples by sequential centrifugation and ultracentrifugation steps at 2,000 × *g* for 10 min at 10 °C to remove debris, followed by centrifugation at 12,000 × *g* for 20 min to pellet large EVs (lEVs). The resulting supernatant was ultracentrifuged at 100,000 × *g* for 70 min to obtain crude small EVs (sEVs, P100), which were washed in PBS and pelleted again under the same conditions. The final pellet was resuspended in PBS and stored at -80 °C. For further purification, iodixanol DG separation was subsequently performed by loading crude sEVs at the bottom of a discontinuous iodixanol gradient followed by ultracentrifugation at 100,000 × *g* for 16 h at 10 °C, after which fractions were sequentially collected from the top of the gradient. Protein annotations and EV/NV classifications used for comparative analyses were derived from the reference dataset^[16]^.

## RESULTS

### PROSPR for the enrichment of EVs from blood

Building upon the established capabilities of the method, we investigated the potential of PROSPR to directly concentrate subsets of EVs from blood and to determine the optimal initial sample volumes. To ascertain the optimal input volume for PROSPR-based enrichment, blood aliquots of 50, 100, and 200 µL were processed, and the resulting EV yield and total protein content were quantified by dFC [Figure 1A and B]. A clear increase in the starting volume resulted in a proportional rise in particle concentration, with the 200 µL condition yielding (5.4 ± 1.1) × 10^8^ particles/mL, significantly higher than both 100 µL (1.6 ± 0.3 × 10^8^ particles/mL; *P* < 0.05) and 50 µL (6.8 ± 1.4 × 10^7^ particles/mL; *P* < 0.05) [Figure 1A]. A similar trend was observed for total protein, which increased from 1.1 ± 0.2 mg/mL at 50 µL to 1.8 ± 0.3 mg/mL at 100 µL (*P* < 0.01) and 3.0 ± 0.5 mg/mL at 200 µL (*P* < 0.001) [Figure 1B]. Linear regression analysis revealed a notably strong correlation between protein concentration and EVs number (*r* = 0.956, *P* = 0.003) [Figure 1C]. The particle-to-protein ratio was also assessed, yielding values of 8.0 × 10^7^, 7.1 × 10^7^, and 1.7 × 10^8^ particles/mg at input volumes of 50 µL, 100 µL, and 200 µL, respectively. All three values substantially exceed the International Society for Extracellular Vesicles (ISEV)/MISEV purity threshold for blood-derived EVs isolates by a considerable margin^[16]^. Consequently, based on the data obtained, a blood input volume of 200 µL was identified as optimal for maximizing EV recovery while also enhancing purity. This volume is also compatible with clinical applications and was thus selected as the standard condition for subsequent PROSPR workflows in this study.

**Figure 1:**
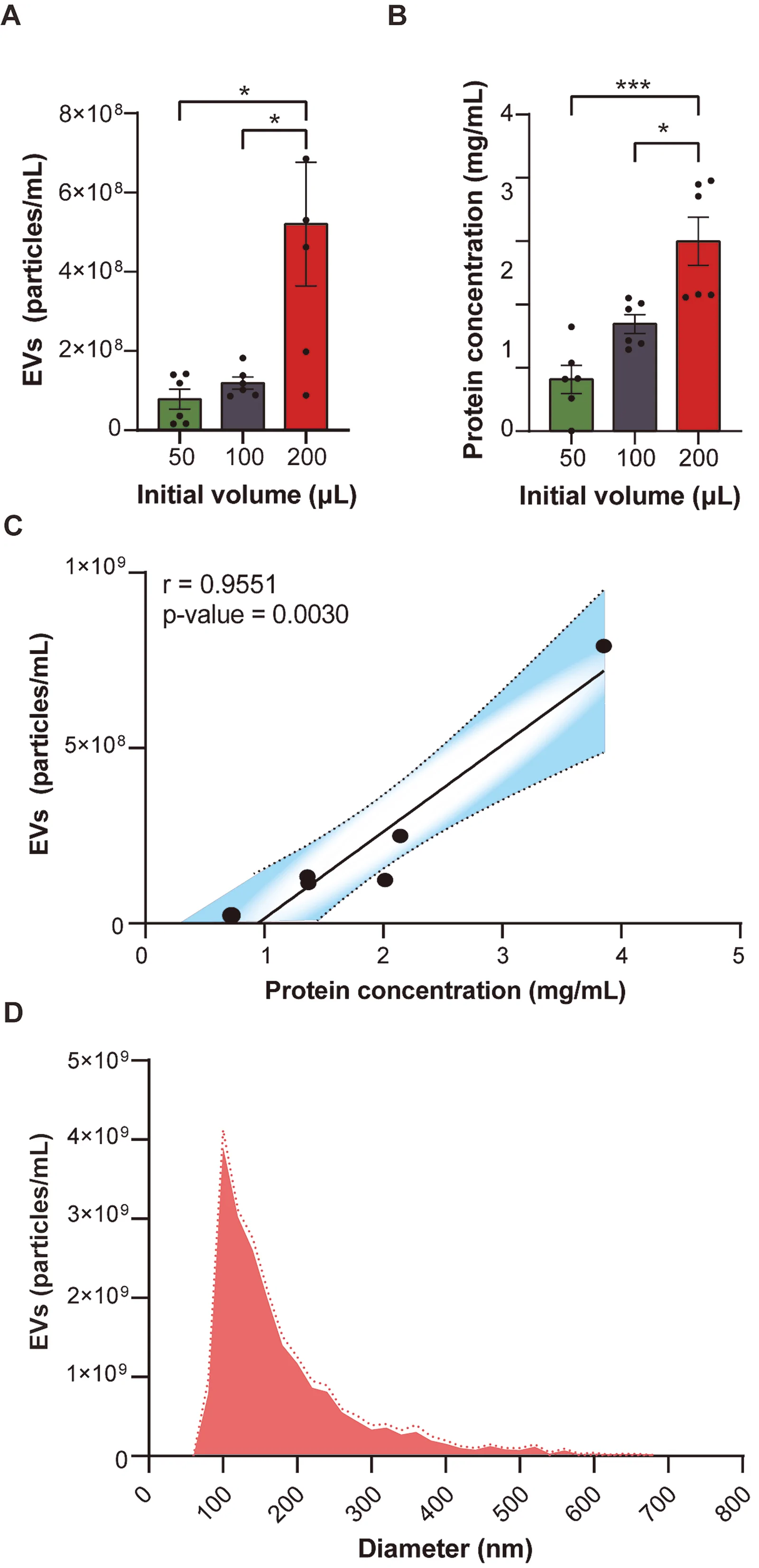
Optimization of the PROSPR protocol for EV enrichment from blood samples. (A) Average EV particle concentration (particles/mL) measured by dFC across varying starting volumes of blood (50, 100 and 200 μL); (B) Average EV protein concentration (mg/mL) quantified by BCA assay for the same blood volumes; (C) Correlation between average EV particle concentration and average protein concentration for each initial blood volume condition. Pearson correlation analysis was applied to evaluate significance; (D) EV size distribution (nm) and particle concentration (particles/mL) obtained by TRPS from PROSPR-enriched EVs from blood. Discontinuous lines indicate SEM. Statistical significance was assessed using one-way ANOVA (^*^P < 0.05, ^**^P < 0.01, ^***^P < 0.001). EV: Extracellular vesicle; dFC: dedicated flow cytometry; BCA: bicinchoninic acid; TRPS: tunable resistive pulse sensing; PROSPR: PRotein Organic Solvent PRecipitation; SEM: standard error of the mean; ANOVA: analysis of variance.

To corroborate the dFC measures obtained, blood PROSPR eluates were subsequently analyzed by TRPS. TRPS registered (9.0 ± 0.9) × 10^8^ particles/mL - a ~1.7-fold increase over the bulk dFC particle count, consistent with the higher sensitivity of TRPS for sub-100 nm vesicles [Figure 1D and Supplementary Table 1]. The modal hydrodynamic diameter was determined to be 181 ± 5 nm, with over 80% of events occurring within the 120-260 nm range [Figure 1D and Supplementary Table 1]. Moreover, the TRPS analysis did not indicate any detectable contamination by proteins or lipoprotein aggregates, as no peaks were observed in the lower range of the distribution. This finding was also supported by the particle-to-protein ratio assessments previously conducted.

### Physicochemical properties of PROSPR-enriched EVs from blood

TEM verified the vesicular morphology of blood EVs, corroborating previous findings from PROSPR-enriched EVs^[13,14]^. Consequently, ultrastructural characterization revealed intact, purified, and spherical bilayer structures with diameters ranging from 100 to 150 nm [Figure 2A]. dFC analysis additionally identified three principal sub-populations [Figure 2B and C]. sEVs (< 150 nm) constituted approximately 10% of the total events, while lEVs (150-500 nm) comprised approximately 70% of total events. Particles exceeding 500 nm accounted for the remaining 20%. Absolute counts reflected these proportions: the intermediate sub-population reached nearly 6 × 10^5^ particles per run, significantly surpassing both the small and large fractions (^**^P < 0.01) [Figure 2B and C].

**Figure 2:**
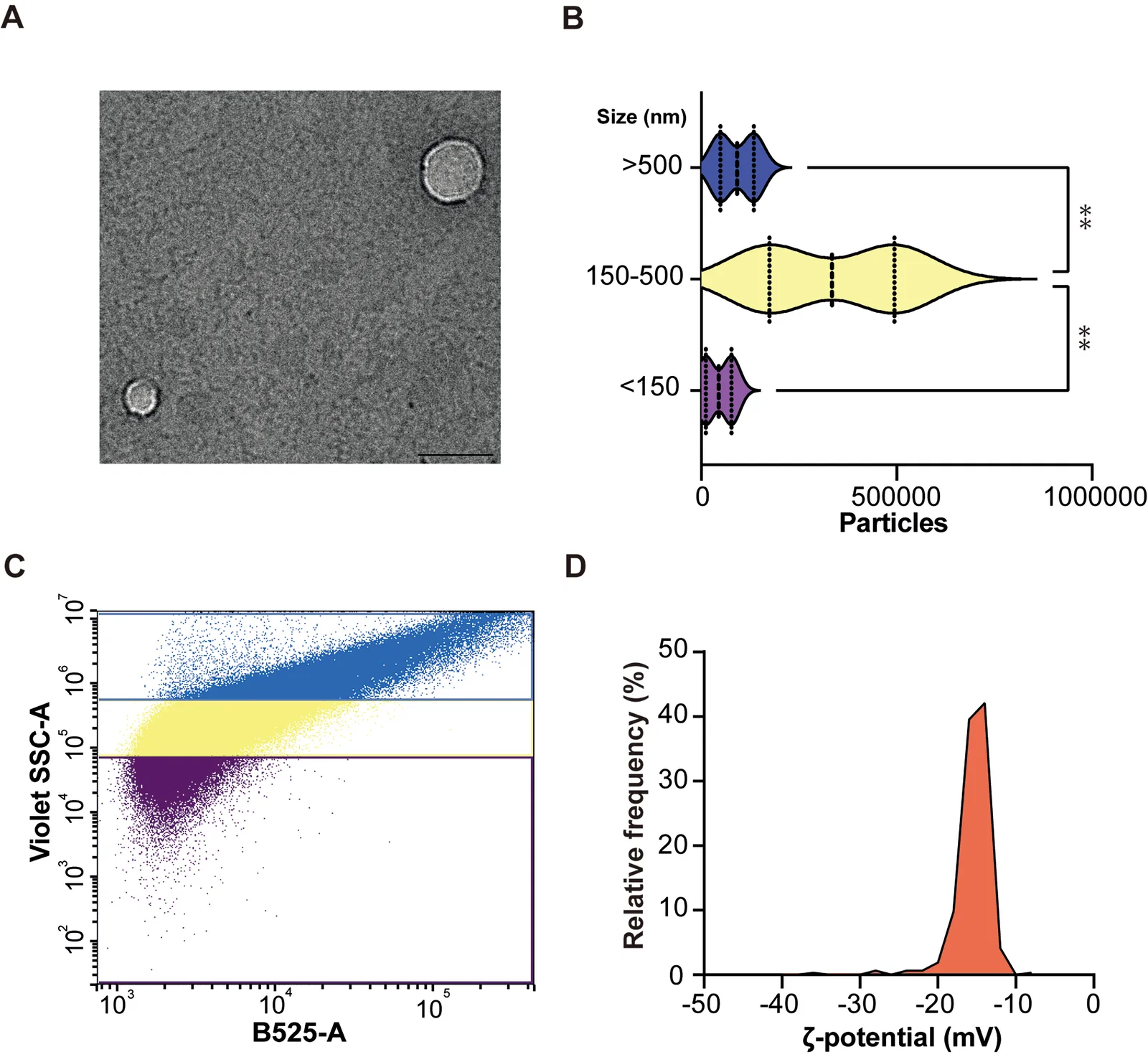
Morphological and physicochemical characterization of EVs isolated from blood using the optimized PROSPR protocol. (A) Representative TEM image of EVs isolated from blood using the PROSPR protocol. Scale bars: 100 nm; (B) Characterization of EV particles by dFC. Particle counts are shown by size categories: < 150 nm (purple), 150-500 nm (yellow), and > 500 nm (blue); (C) Quantitative size distribution of blood-derived EVs measured by dFC; (D) Electrophoretic Zeta potential (ζ-potential) distribution of blood-derived EVs measured by TRPS. The histogram shows the relative frequency of particles as a function of ζ-potential (mV). Data represent a single homogeneous population with a mean ζ-potential of -15.3 ± 1.8 mV. Statistical significance was defined as *P* < 0.05. Asterisks denote levels of significance (^**^P ≤ 0.01). Statistical differences were assessed by one-way ANOVA. EVs: Extracellular vesicles; TEM: transmission electron microscopy; dFC: dedicated flow cytometry; TRPS: tunable resistive pulse sensing; PROSPR: PRotein Organic Solvent PRecipitation; ANOVA: analysis of variance.

Further characterization of blood EVs was performed using TRPS, which enabled particle-by-particle measurements of hydrodynamic diameter and electrophoretic mobility (ζ-potential). This methodology enabled a quantitative evaluation of the surface charge and aggregation tendency of the vesicles within the obtained EVs-enriched preparations. The analyses, as expected, indicated that blood PROSPR-enriched vesicles exhibit a consistently moderate negative surface charge [Figure 2D and Supplementary Table 2]. These vesicle preparations demonstrated a mean ζ-potential of -15.3 ± 1.8 mV across all biological and technical replicates performed (*n* = 3 biological replicates, with three technical replicates) [Figure 2D and Supplementary Table 2], thereby confirming the presence of a single, homogeneous population suggesting no detectable highly charged debris.

### Comparative study of PROSPR-enriched EVs from blood and plasma fractions

TEM ultrastructural study confirmed that PROSPR enriches a population predominantly composed of sEVs from both blood and plasma matrices, as illustrated in Figure 3A and B, respectively. EVs from both matrices retained the same canonical morphology - preserved bilayered, cup-shaped vesicles - and exhibited negligible frequencies above 200 nm [Figure 3A and B]. We then performed ultra-sensitive TRPS characterization to assess whether PROSPR-enriched vesicles from blood and plasma provide complementary size fractions, thereby offering a more comprehensive representation of the circulating EV repertoire in blood for downstream molecular analyses. These analyses underscored a matrix-dependent shift in absolute particle numbers [Figure 3C and D, Supplementary Table 3]. However, it was noted that the two distributions largely overlapped, with both peaking at approximately 170 nm and tapering off by around 350 nm [Figure 3C]. The mean diameter of the blood-derived EVs was 173 ± 4 nm, in contrast to 163 ± 3 nm for those derived from plasma, a difference that was statistically significant (*P* = 0.025) [Figure 3D]. TRPS also revealed, through the analysis of the ζ-potential in blood and plasma enriched EVs, that the 95% confidence interval for the mean difference included zero [Figure 3E and Supplementary Table 4]. The overlapping distributions centered around -15 mV [Figure 3E], thereby substantiating the existence of a singular, homogeneous population devoid of highly charged debris.

**Figure 3:**
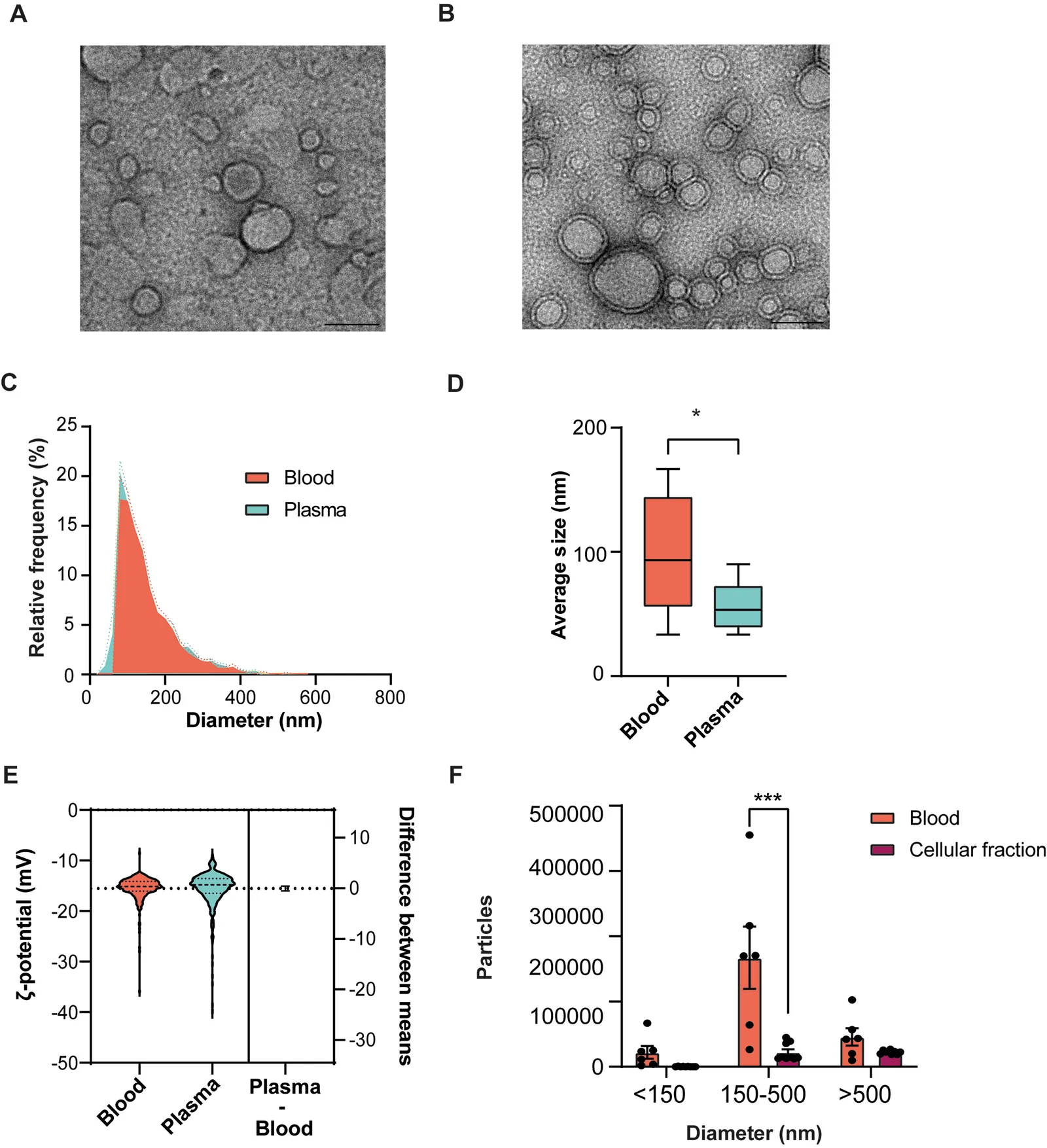
Morphological characterization of PROSPR-enriched EVs from blood, plasma, and from the plasma-depleted blood fraction (cellular fraction) obtained after plasma separation. (A) Representative TEM micrograph of EVs enriched from blood; (B) Representative TEM micrograph of EVs enriched from plasma. Scale bars represent 100 nm in all TEM panels; (C) Histogram of the relative frequency of EV diameters measured by TRPS in blood (red) and plasma (blue). Discontinuous lines indicate the SEM; (D) Quantitative size distribution (mean diameter) of EVs from blood and plasma measured by TRPS; (E) Estimation plot comparing the ζ-potential between blood and plasma measured by TRPS; (F) Quantitative comparison of EV particle count size distribution from blood (red) and cellular fraction (dark red) measured by dFC. Particle counts are represented by size categories: < 150 nm, 150-500 nm, and > 500 nm. Statistical significance was evaluated using an unpaired *t*-test and considered at *P *< 0.05, represented as follows: ^*^P ≤ 0.05; ^***^P ≤ 0.001; ns indicates no significant difference. Analyses were performed using independent biological samples (*n* = 3 per condition), each with three technical replicates. PROSPR: PRotein Organic Solvent PRecipitation; TEM: transmission electron microscopy; EVs: extracellular vesicles; TRPS: tunable resistive pulse sensing; SEM: standard error of the mean; dFC: dedicated flow cytometry.

Furthermore, we assessed the impact of plasma removal on the vesicle size distribution within the cellular fraction. dFC analyses identified a concentration of PROSPR-enriched EVs from blood predominantly within the 150-500 nm range, yielding approximately 4 × 10^5^ particles per run. This is significantly greater than the count observed in the cellular fraction, which is approximately ~5 × 10^4^ particles (*P* < 0.0001) [Figure 3F]. Notably, there were no identified significant differences in the < 150 nm or > 500 nm subpopulations between the two matrices. This finding suggests that plasma separation preferentially depletes EVs in the intermediate size range, while smaller and larger vesicles are largely retained in the remaining cellular fraction. Consequently, these results emphasize the value of analyzing both cellular fraction and plasma to achieve a comprehensive understanding of the blood circulating EV repertoire.

### High-resolution lipidomics of PROSPR-enriched EVs from blood and plasma

Untargeted LC-ESI-Q-TOF analysis of PROSPR-enriched EVs from blood and plasma identified 1,599 lipid species spanning 16 structural families [Figure 4A and Supplementary Table 5]. Among these, 189 species were identified as significantly regulated after adjusting for the false discovery rate (FDR), resulting in 1,410 non-significant entries that were common across the analyzed conditions [Figure 4B]. PCA effectively distinguished PROSPR-enriched EV lipidomes based on their matrix origin. Blood EV samples formed a compact cluster on the negative side of Dim1, accounting for 30% of the variance, whereas plasma EV samples exhibited a more dispersed cluster on the positive side of Dim1, indicating a distinct compositional shift [Figure 4C]. Hierarchical clustering of the most discriminating lipids consistently identified two distinct clusters corresponding to the blood versus plasma grouping [Figure 4D]. Despite this differentiation, the class-summation profiles demonstrated broad similarities, with both matrices predominantly composed of neutral lipids, particularly triradylglycerols (TGs) [Figure 4D]. However, specific quantitative differences were noted, most notably a relative enrichment in blood EVs of lipid features putatively assigned to phosphatidylserine or phosphatidylethanolamine (PS/PE), as well as sphingomyelin or ceramide-1-phosphate (SM/CerP) species, whereas plasma EVs showed a higher relative abundance of triglycerides [Figure 4D]. Similarly, an analysis of the lipid family distribution revealed that TG constituted the majority of the total ion intensity in both blood- and plasma EVs; however, at the lipid family level, these remained unchanged within analytical variation [Figure 4E-G].

**Figure 4:**
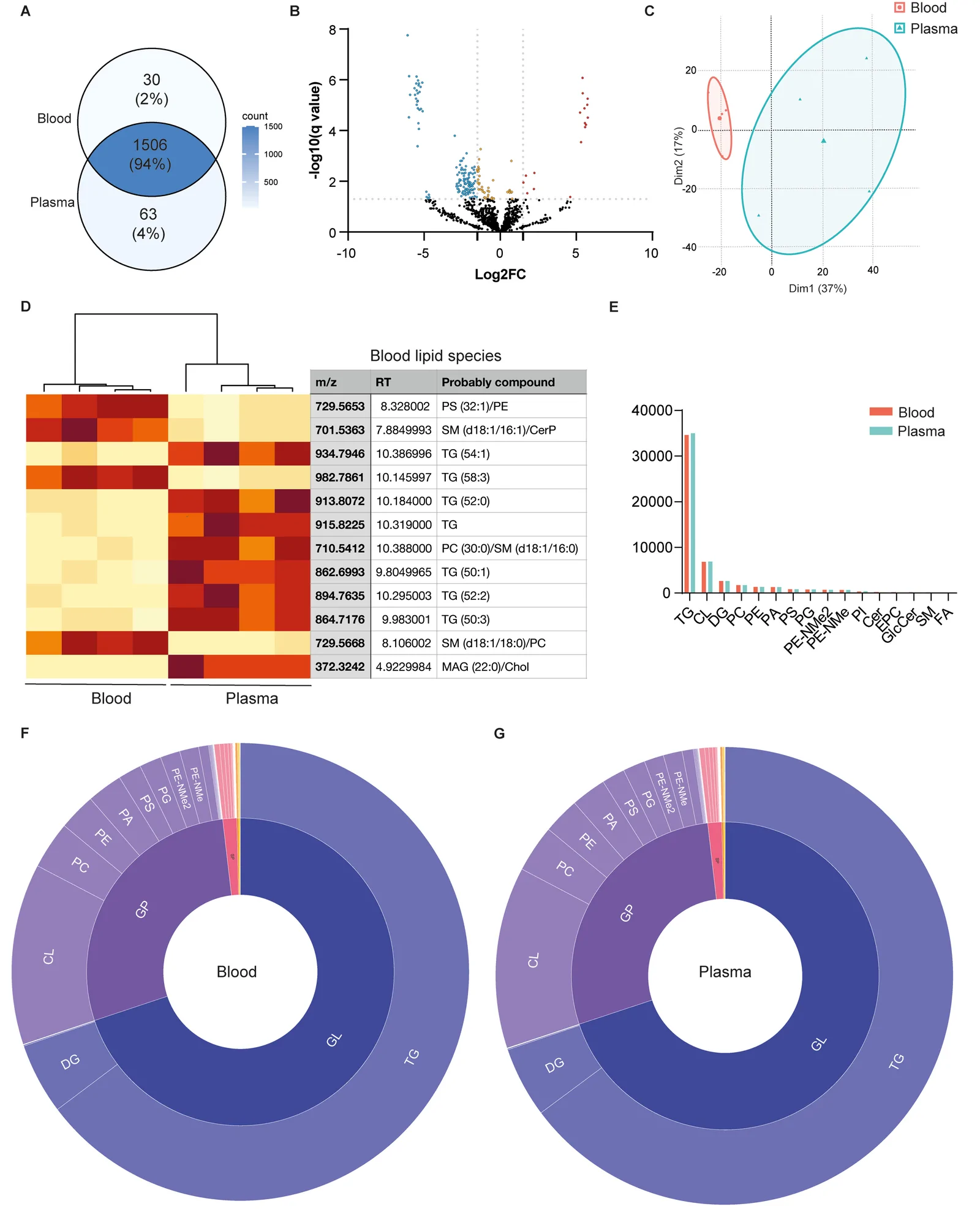
Untargeted lipidomic comparison between EVs isolated from blood and plasma using the PROSPR enrichment protocol. (A) Venn diagram showing the number of lipid species identified by untargeted lipidomics in blood and plasma EVs; (B) Volcano plot displaying the differential abundance of lipid species between blood and plasma EVs. Lipids with significantly higher abundance in blood EVs compared to plasma (log_2_FC > 1.5, *P* < 0.05) are shown in red; those with significantly lower abundance in blood EVs (log_2_FC < -1.5, *P* < 0.05) in blue; lipids with statistically significant but smaller fold changes (|log_2_FC| < 1.5) in orange; and non-significant lipids (*P* ≥ 0.05) in black; (C) PCA based on lipidomic profiles of blood (red) and plasma (blue) EVs, revealing sample group separation; (D) Heatmap and hierarchical clustering of the most discriminating lipid species between blood and plasma EVs. Clustering was performed using Euclidean distance and Ward’s linkage method. In cases where multiple lipid classes are shown as a putataive compound, the detected feature could not be unambiguously assigned to a single lipid species; (E) Quantification of lipid classes identified in blood (red) and plasma (blue) EVs; (F) Circular plot showing the lipid family and subclass composition of lipid species identified in blood EVs; (G) Circular plot showing the lipid family and subclass composition of lipid species identified in plasma EVs. In F and G, the inner ring denotes the main lipid family; the outer ring indicates specific lipid subclasses. Statistical significance was evaluated using unpaired *t*-test (*P* < 0.05). EVs: Extracellular vesicles; PCA: principal component analysis; GL: glycerolipids; GP: glycerophospholipids; SP: sphingolipids; TG: triradylglycerols; CL: cardiolipin; DG: diacylglycerols; PC: phosphatidylcholine; PE: phosphatidylethanolamine; PA: phosphatidic acid; PS: phosphatidylserine; PG: phosphatidylglycerol; PE-NMe_2_: dimethyl phosphatidylethanolamine; PE-NMe: monomethyl phosphatidylethanolamine; PI: phosphatidylinositol; Cer: ceramides; EPC: ether-linked phosphatidylcholine; GlcCer: glucosylceramide; SM: sphingomyelin; FA: fatty acids; MAG: monoacylglycerol; Chol: cholesterol.

### High-depth proteomics of PROSPR-enriched EVs from blood and plasma

Label-free proteomics analysis identified 1,790 non-redundant proteins across all PROSPR-enriched plasma and blood EVs examined [Figure 5A and Supplementary Table 6]. The majority of these proteins were detected exclusively in plasma EVs (1,337; 75%), while 251 (14%) were unique to blood EVs, and 202 (11%) were shared, highlighting the matrix-specific complementarity of the two PROSPR workflows. To benchmark these proteomes against established EV signatures, we compared them with the top 100 proteins curated in the specialized databases ExoCarta and Vesiclepedia [Figure 5B and C]. Seventy-five of the top 100 ExoCarta markers were identified: 41 were present in EVs from both matrices, 16 were exclusive to blood EVs, and 18 were exclusive to plasma EVs. Coverage was even higher for Vesiclepedia, with 85 markers detected (43 common, 17 blood-specific, 25 plasma-specific) [Figure 5B and C]. Thus, while plasma EVs provide broader overall proteome depth, the blood contributes unique EV proteins - including canonical markers - thereby expanding the detectable vesicle proteome when both fractions are analyzed in tandem.

**Figure 5:**
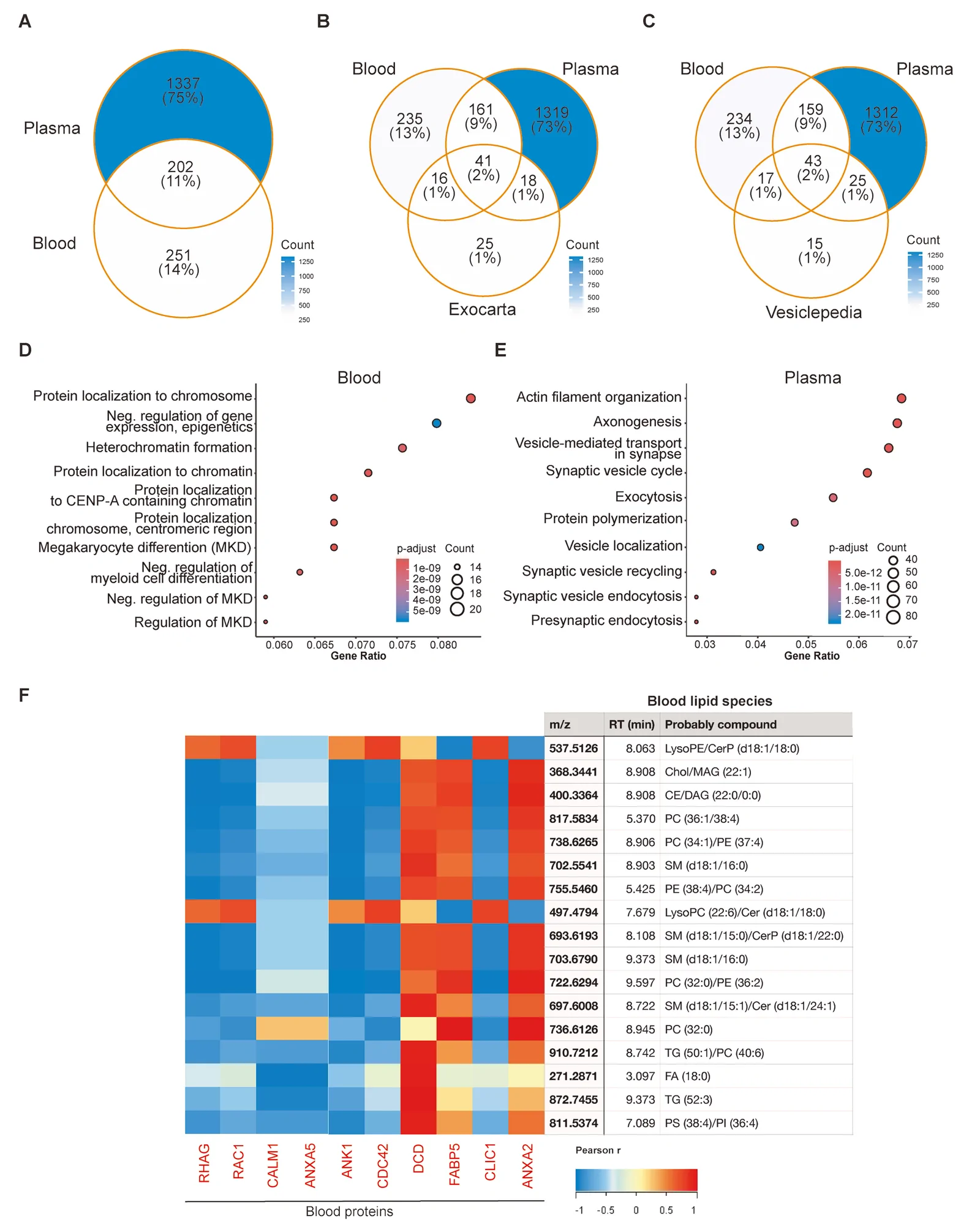
Proteomic and multi-omic characterization of PROSPR-enriched EVs from blood and plasma. (A) Venn diagram showing the number of proteins identified in blood and plasma-derived EVs; (B) Overlap of identified proteins with the top 100 most common EV-associated proteins listed in the ExoCarta database; (C) Overlap of identified proteins with the top 100 proteins in the Vesiclepedia database; (D) Functional enrichment analysis of biological processes associated with proteins exclusively identified in blood-derived EVs. The list of proteins involved in each pathway is listed in Supplementary Table 9; (E) Equivalent functional enrichment analysis for proteins exclusively detected in plasma-derived EVs. Dot size represents the number of proteins per functional category, and color indicates the adjusted *P*-value for enrichment. The list of proteins involved in each pathway is listed in Supplementary Table 10; (F) Multi-omic integration of proteomic and lipidomic profiles of blood-derived EVs. The network shows correlations between membrane-associated proteins and lipid species exclusively present in blood-derived EVs. Pearson correlations (*r* > 0.5) are shown in red, and negative correlations (*r* < -0.5) in blue. Analyses were performed using independent biological samples. PROSPR: PRotein Organic Solvent PRecipitation; EVs: extracellular vesicles; RHAG: Rh-associated glycoprotein; RAC1: Rac Family Small GTPase 1; CALM1: calmodulin 1; ANXA5: annexin A5; ANK1: ankyrin 1; CDC42: cell division cycle 42; DCD: dermcidin; FABP5: fatty acid binding protein 5; CLIC1: chloride intracellular channel protein 1; ANXA2: annexin A2; MKD: megakaryocyte differentiation; RT: retention Time; LysoPE: lysophosphatidylethanolamine; CerP: ceramide Phosphate; Chol: cholesterol; MAG: monoacylglycerol; CE: cholesteryl ester; DAG: diacylglycerol; LysoPC: lysophosphatidylcholine; FA: fatty acid; PC: phosphatidylcholine; SM: sphingomyelin; PE: phosphatidylethanolamine; Cer: ceramides; PS: phosphatidylserine; TG: triradylglycerols.

Pathway-enrichment analysis of the pathways common to both blood and plasma EVs revealed a diverse functional repertoire extending beyond traditional angiogenic and immune pathways associated with chronic disorders including dementia [Table 1, Supplementary Tables 7 and 8]. In addition to the prominent vascular endothelial growth factor A-vascular endothelial growth factor receptor 2 (VEGFA-VEGFR2) axis, severe acute respiratory syndrome coronavirus 2 (SARS-CoV-2) host-response module, complement cascade, and phosphatidylinositol 3-kinase -protein kinase B (PI3K-Akt) signaling, the shared set included: (i) nuclear-receptor networks and multiple cancer-related pathways, indicating a conserved role in hormone and growth regulation; (ii) mitochondrial homeostasis nodes, such as the oxidative phosphorylation electron-transport chain (OXPHOS), Parkin-ubiquitin proteasomal quality control, and histone deacetylase 6 (HDAC6) interactions in the central nervous system, highlighting shared connections to bioenergetics and organelle turnover; (iii) infection and coagulation interfaces, including complement-coagulation cross-talk; and (iv) pathways associated with cellular stress and adhesion [focal-adhesion, mitogen-activated protein kinase (MAPK), glycolysis/gluconeogenesis] [Table 1, Supplementary Tables 7 and 8]. In contrast, the proteome subsets unique to each matrix demonstrated significantly divergent functional themes. Proteins specific to blood EVs were enriched in distinct chromatin-associated processes, including protein localization to chromosomes, heterochromatin organization, and nucleosome assembly, as well as megakaryocyte differentiation [Figure 5D and Supplementary Table 9]. Conversely, proteins specific to plasma EVs were associated with neuronal and cytoskeletal pathways, such as synapse organization, actin-filament dynamics, axonogenesis, and synaptic-vesicle cycling [Figure 5E and Supplementary Table 10].

**Table 1:** Biological pathways commonly identified in the proteomes of PROSPR-enriched EVs derived from blood and plasma. Pathways detected in both EV populations are listed, together with the number of proteins assigned to each pathway in the corresponding blood- and plasma-derived EV proteomes.

**Pathway**	**Blood**	**Plasma**
VEGFA-VEGFR2 signaling	21	39
Complement system	17	24
Alzheimer’s disease	15	22
Alzheimer’s disease and miRNA effects	15	22
Complement and coagulation cascades	14	17
Metabolic epileptic disorders	12	19
Vitamin D receptor pathway	12	5
Network map of SARS-CoV-2 signaling	11	27
Selenium micronutrient network	11	13
Glycolysis and gluconeogenesis	11	11
Clear cell renal cell carcinoma pathways	11	10
Vitamin B12 metabolism	10	13
Folate metabolism	10	11
16p11.2 proximal deletion syndrome	10	7
Proteasome degradation	10	5
Ras signaling	10	5
Nuclear receptors meta-pathway	9	25
Metabolic reprogramming in colon cancer	9	9
Cancer pathways	8	23

PROSPR: PRotein Organic Solvent PRecipitation; EVs: extracellular vesicles; VEGFA-VEGFR2: vascular endothelial growth factor A-vascular endothelial growth factor receptor 2; miRNA: microRNA; SARS-CoV-2: severe acute respiratory syndrome coronavirus 2.

### Integrated multi-omics profiling of PROSPR enriched EVs from blood

The integration of lipidomic and proteomic datasets has elucidated matrix-specific molecular interactions that remain undetectable through single-omic analyses. A comprehensive correlation heat map, constructed from blood-exclusive EV proteins and blood-exclusive EV lipids, identified two coherent modules [Figure 5F]. One module demonstrated positive correlations (*r *> 0.5, red) between Dermcidin (DCD) and neutral lipids such as TGs, while the other module exhibited mirroring negative correlations (*r* < -0.5, blue) between annexin and membrane-associated proteins [e.g., annexin A5 (ANXA5), rhesus blood group-associated glycoprotein (RHAG)] and polar lipids such as phosphatidylserines (PSs) or phosphatidylinositols (PIs), sphingomyelins (SMs) or ceramides (Cers) [Figure 5F].

### Benchmarking PROSPR blood-derived EVs against gold-standard ultracentrifugation

To further assess the efficacy of PROSPR in comparison to established EV enrichment techniques, we conducted a comparative analysis of PROSPR-enriched EVs derived from blood against EV preparations obtained via dUC followed by DG (dUC-DG), a method widely recognized as a gold standard in the field. Proteomic datasets from both methodologies were systematically analyzed to evaluate the specificity of EV enrichment and the extent of contaminant co-isolation, accounting for differences in starting biofluid matrices between workflows.

A comparative overlap analysis demonstrated that, although each method uniquely identified a subset of proteins, a common core proteome was consistently detected across both workflows, as illustrated in Figure 6A. Specifically, 168 proteins were common to both methods, whereas 574 proteins were uniquely identified in dUC-DG preparations, and 285 proteins were exclusive to PROSPR-enriched EVs. This distribution underscores both the complementarity and the method-specific biases inherent to each isolation strategy.

**Figure 6:**
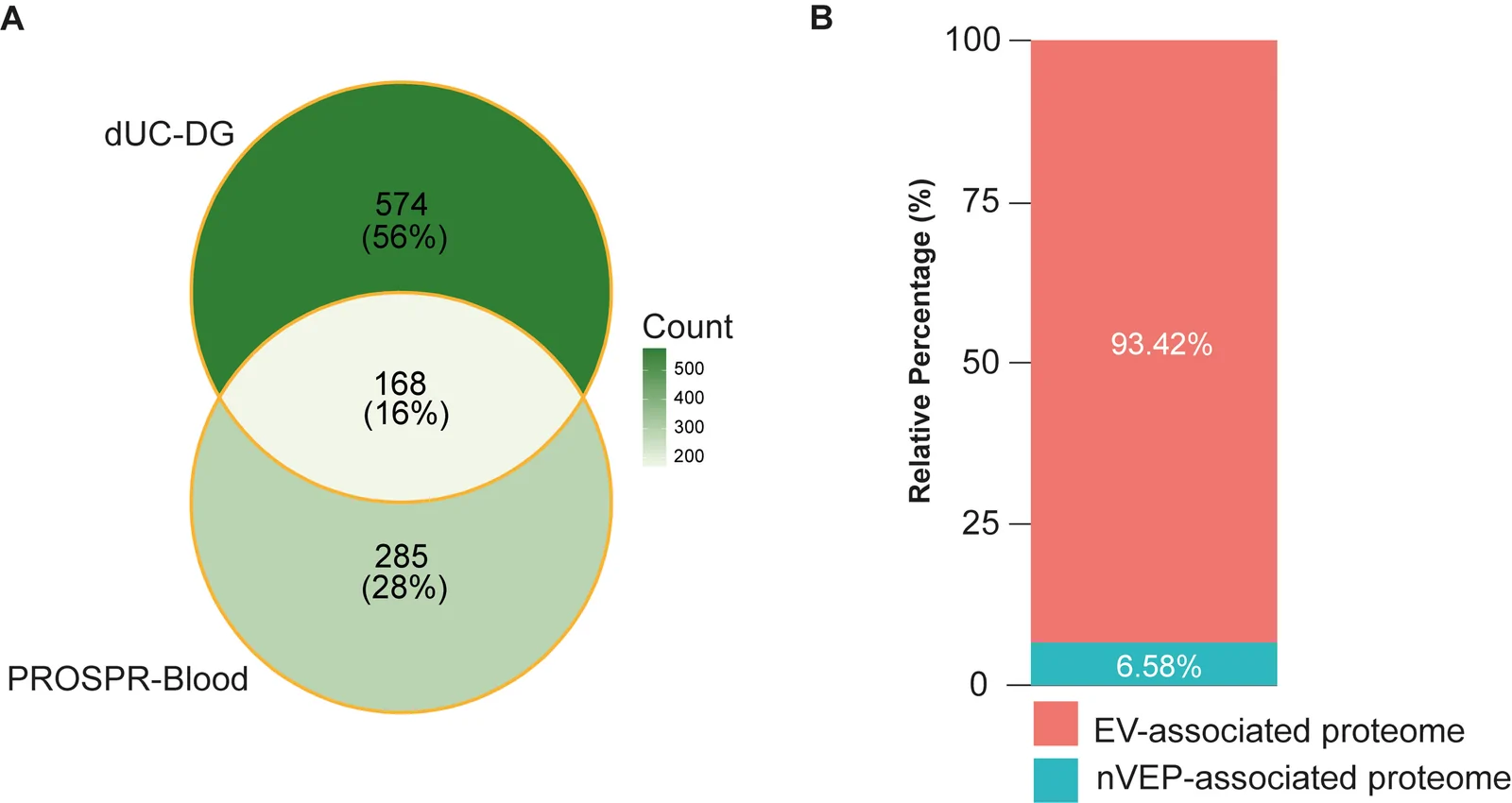
Comparative and internal enrichment analyses of PROSPR blood-derived EV preparations and contaminant-associated protein contribution. (A) Venn diagram showing the overlap between proteins identified in PROSPR-enriched EVs and EV preparations obtained by dUC-DG. The analysis identified a shared core of 168 proteins, with 285 PROSPR-specific and 574 dUC-DG-specific proteins, highlighting both common EV-associated features and method-dependent proteomic signatures. Comparative benchmarking analyses were performed using the protein categorization approach applied in the reference dataset reported by Morales-Sanfrutos *et al.*^[16]^; (B) Relative distribution of EV-associated proteins and nVEPs in PROSPR-enriched blood EV preparations according to MISEV-based protein classification criteria^[17]^. EV-associated proteins accounted for 93.42% of the total proteomic signal, whereas non-EV proteins represented 6.58%, indicating low contaminant contribution in PROSPR-enriched samples. PROSPR: PRotein Organic Solvent PRecipitation; EV: extracellular vesicle; dUC-DG: differential ultracentrifugation followed by density gradient; nVEPs: non-vesicular extracellular proteins; MISEV: Minimal Information for Studies of Extracellular Vesicles.

To comparatively evaluate EV enrichment relative to co-isolated contaminants across enrichment workflows, comparative log2 enrichment ratios were calculated based on the relative abundance of EV-associated versus nVEP-associated proteins shared between datasets. Protein classifications were assigned according to the categorization approach applied in the comparative dataset reported by Morales-Sanfrutos *et al.*^[16]^. This comparative benchmarking analysis yielded enrichment ratios of -3.11 for dUC-DG and -1.92 for PROSPR. As the enrichment statistic is defined as log2 (EV-associated signal/nVEP-associated signal), less negative values indicate a higher EV-to-contaminant ratio and therefore a lower relative contribution of contaminants. Accordingly, PROSPR-enriched blood EV preparations showed a lower contaminant signal compared with dUC-DG.

Importantly, an independent bootstrap-based internal validation analysis was subsequently performed exclusively on the PROSPR dataset using nVEP-associated protein categories defined according to the updated MISEV guidelines^[17]^. This analysis revealed that EV-associated proteins accounted for 93.42% of the total proteomic signal, whereas nVEP-associated proteins represented only 6.58% [Figure 6B]. The bootstrap-derived EV enrichment statistic showed a robust positive distribution, with a 95% confidence interval ranging from 2.02 to 8.16 [Supplementary Table 11]. Because the MISEV-based categorization focuses on canonical contaminant-associated protein groups and is less stringent than the broader classification framework applied in the comparative benchmarking analysis, the resulting enrichment estimates are not directly equivalent between the two approaches. These findings support a consistently low contribution of nVEP-associated proteins and reinforce the effective enrichment of bona fide EV-associated proteomic content.

Collectively, these results indicate that PROSPR achieves EV enrichment efficiencies comparable to conventional ultracentrifugation-based workflows, despite differences in the starting biological matrices, while also providing a robust and internally validated reduction in contaminant contribution and capturing a distinct subset of the circulating EV proteome.

## DISCUSSION

In this study, we demonstrate, for the first time, that an optimized PROSPR workflow enables the direct enrichment, quantitative characterization, and multi-omics interrogation of EVs from blood. By coupling PROSPR with dFC, TRPS, TEM and high-depth mass spectrometry based lipidomic-proteomic pipelines, we show that (i) a 200 µL input volume is sufficient to recover highly pure EV preparations that span the canonical nano-to micro-vesicle continuum; and (ii) vesicle integrity is preserved - ζ-potentials remain within the expected -10 to -30 mV window and single-particle blockade histograms are unimodal. In comparison to other standard workflows for the enrichment of EVs, it is noteworthy that the PROSPR method enables enrichment in less than 20 min using a benchtop centrifuge. Given that blood is routinely archived in biobanks and, in certain instances, constitutes the sole clinical material available from valuable cohorts - yet is infrequently utilized for EV studies^[18,19]^ - the present optimized workflow offers a standardized and readily implementable approach to unlock this dormant resource for biomarker discovery. This is particularly significant given the importance of early detection, disease prediction, and mechanistic research associated with EVs. Furthermore, the workflow delineated in this study can be effectively integrated into clinical settings, given its minimal demands on time and equipment, a factor of considerable significance.

TRPS, dFC, and TEM analyses corroborated that PROSPR application to blood enriches EVs within the 120-260 nm range. This range coincides with - and slightly extends - the dominant sEVs population described by Arraud *et al.*^[20]^, supporting that the method selectively enriches sEVs while retaining the lower end of the lEVs continuum. The authors established that approximately 80% of circulating vesicles in serum and plasma profiles are below the 300 nm threshold^[20]^, consistent with our observations.

Remarkably, our data further indicated that no significant contamination was detected in PROSPR-enriched EVs preparations derived from blood, as determined through ultrastructural analyses. Moreover, the protein-to-nanoparticle ratios obtained consistently met or exceeded the MISEV purity standards for plasma and serum circulating EVs^[16]^. PROSPR EVs, characterized using TRPS, an advanced technique that measures ionic current reduction as particles traverse a calibrated nanopore, thereby providing ζ-potential proxies for vesicles and aggregates^[21]^. These EVs exhibited moderately negative ζ-potentials within the appropriate range. This finding indicates the preservation of mammalian lipid bilayers and also points to the absence of lipoprotein aggregates and relevant soluble interacting lipoproteins attached to the membranes^[22]^. Understanding particle surface charge is essential for evaluating the quality of EV preparations, as it influences vesicle purity, stability, protein interactions, and cellular uptake, thereby affecting the diagnostic and therapeutic efficacy of the subsequent analyses^[23-25]^. Moreover, to the best of our knowledge, this study provides the first evidence that the detergent-free PROSPR workflow maintains EV membrane integrity despite the central use of organic solvent^[13,14]^. This finding, linked to previous optimizations of the method for plasma and solid tissues^[13,14]^ holds significant implications for its potential application in subsequent functional assays.

Building upon these biophysical observations, our study further confirmed that PROSPR-enriched EVs, isolated directly from blood, are suitable for comprehensive systems-biology-scale assays without the requirement of any additional purification steps. This compatibility is crucial for biomarker discovery and mechanistic investigation of circulating EVs, as reviewed by Shaba *et al.*^[26]^. Therefore, advanced untargeted lipidomics^[27]^ revealed that PROSPR workflow maintains distinct lipid profiles characteristic of the original matrix of EVs, despite overall similarities in lipid family compositions between blood and plasma samples. Both matrices showed a predominance of neutral lipids, particularly TGs, highlighting fundamental compositional similarities. However, significant quantitative differences were identified at the species level. Blood-derived EVs exhibited relative enrichment of specific PS/PE and SM/CerP species, suggesting a prominent contribution of platelet- and mitochondria-derived vesicles, consistent with previous studies^[28,29]^. Conversely, plasma EVs displayed a notable increase in specific TGs, potentially indicative of hepatocyte- and endothelial-related origins^[30]^. Notably, the preservation of these subtle, matrix-dependent lipidomic differences observed suggests that PROSPR enrichment does not cause artifactual lipid exchange or loss, corroborating our above detailed biophysical data. This is a crucial prerequisite for the accurate modeling of downstream pathways. PCA and hierarchical clustering further supported these findings, clearly separating EV lipidomes by matrix origin, with blood-derived samples forming tight, cohesive clusters distinct from the more dispersed plasma EV lipidome profiles.

LC-MS/MS in-depth proteomic analyses also underscored the complementary nature of PROSPR isolates derived from blood-related matrices. While plasma EVs offered a broader analytical scope, blood yielded unique proteome identifications, including vesicle markers that were not detectable in plasma-only workflows. More than 200 proteins common to both matrices formed a “core” signature, which was enriched in VEGFA-VEGFR2, complement, SARS-CoV-2 host-response, and PI3K-Akt modules - pathways that are associated with EV-mediated vascular homeostasis and innate immunity. The integration of both datasets expanded the coverage to over 80% of the ExoCarta/Vesiclepedia top-100 list of vesicle markers, while elucidating matrix-specific functions - such as chromatin and megakaryocyte-differentiation pathways in blood, and synaptic and cytoskeletal remodeling in plasma. While these state-of-the-art omics analyses clearly demonstrate that the dual-matrix approach remains optional, our findings strongly indicate that complementary analysis can enhance biological resolution^[31]^. Furthermore, blood PROSPR preparations represent a robust resource for biomarker discovery and the identification of relevant health- and disease-associated pathways in circulating EVs.

Finally, our multi-omics integrative analyses, contributed to identify a coordinated cargo-sorting mechanism^[32]^ that is obscured when blood is processed into plasma. Correlation mapping has revealed mirrored blocks of associations: a platelet-scaffold protein such as ANXA5 is co-packaged with curvature-promoting lysophosphatidylcholine (LPC) and PE species, whereas these lipids are depleted in the absence of the scaffolds^[33]^. This observation suggests that vesicles derived from megakaryocytes or activated platelets assemble lipid micro-domains that stabilize curved, pro-adhesive membranes - features that are lost during plasma preparation. Pathway enrichment analysis of these proteins [RAS-mitogen activated protein kinases (RAS-MAPK), VEGFA-VEGFR2, Ca^2+^ signaling, DNA-damage responses] indicates an integrated signaling platform where membrane receptors, cytoskeletal anchors, and regulatory lipids facilitate vascular repair and immune surveillance^[34]^. Plasma-exclusive vesicles retain synaptic and metabolic signatures, underscoring the complementary information present in both matrices. The integration of multi-omics data confirms that analyzing PROSPR-enriched EVs from blood and plasma provides the most comprehensive insights, while blood fraction offers unique diagnostic and mechanistic cues.

A potential consideration in this study is the complexity of blood as starting material. Blood contains platelets, erythrocytes, leukocytes and subcellular components that may contribute vesicular and non-vesicular material during sample processing. The isolation strategy applied here has demonstrated suitability for EV enrichment in complex biological matrices, including adaptations to brain tissue, and has been used in multiple protocols for downstream targeted systems biology approaches. However, we cannot fully exclude that some platelet- or mitochondria-associated signals may partly originate from *ex vivo* platelet activation, limited cellular damage, or co-isolation of cellular debris and organelle-derived fragments, rather than exclusively from circulating EVs present *in vivo*. We therefore interpret these signals with appropriate caution. This should be viewed as an inherent challenge of assigning specific molecular cargoes to EVs in complex biofluids, rather than as a limitation of the isolation method itself. Whenever possible, orthogonal validation using spatially resolved or single-particle approaches will be valuable to confirm the direct association of selected molecules of interest with EVs and to support their biological origin *in vivo*.

In addition, although EDTA was used in a standardized manner during processing of the cellular fraction, its specific impact on EV extraction efficiency, vesicle integrity, and downstream molecular profiles was not directly assessed in this study. Therefore, we cannot exclude that EDTA may have contributed, at least in part, to some of the effects observed after plasma removal. If this specific effect is of particular interest, the contribution of EDTA should be directly evaluated under controlled experimental conditions.

In summary, the optimized blood PROSPR workflow presents a swift and reliable technique for extracting EVs from minimal volumes of stored blood, thereby unlocking new possibilities for leveraging valuable biobank collections in EV-based biomarker research. This approach maintains the structural integrity of EVs and generates samples that are ready for comprehensive proteomic and lipidomic analyses without the need for further purification. Significantly, EVs obtained from blood offer molecular insights that plasma EVs alone do not fully capture, including unique proteomic and lipidomic profiles specific to the matrix. Therefore, whole-blood PROSPR acts as a supplementary method to plasma EV analysis, broadening biological resolution and enhancing the exploration of pathways associated with circulating EVs.

## DECLARATIONS

### Availability of data and materials

All omics data generated in this study have been deposited in specialized public repositories to support transparency, reproducibility, and compliance with FAIR data principles. Proteomics data generated in this study have been deposited with the ProteomeXchange Consortium via the PRIDE partner repository under the dataset identifier PXD066173. The metabolomics data generated in this study have been deposited to the EMBL-EBI MetaboLights database and are publicly accessible under the submission identifier MTBLS12801 (Project Title: Characterization of Whole Blood EVs Using Protein Organic Solvent Precipitation). For the comparative analysis with ultracentrifugation gradient-based EV proteomics, previously published proteomics datasets were accessed through the ProteomeXchange Consortium via the PRIDE partner repository under the dataset identifiers PXD062547 and PXD062477. All additional data supporting the findings of this study are included within the manuscript and its supplementary information files or can be obtained from the corresponding author upon reasonable request.

### AI and AI-assisted tool statement

During the preparation of this manuscript, the AI tool Paperpal (version 4.16.5, released 2026-02) was used solely for language editing and textual refinement. Additionally, ChatGPT (version 5.5, released 2026-04) was utilized to assist in the creation and refinement of certain images included in the graphical abstract. However, all final artwork, design, vectorization, and assembly were manually executed by the authors using Adobe Illustrator. The tools did not influence the study design, data collection, analysis, interpretation, or the scientific content of the work. All authors take full responsibility for the accuracy, integrity, and final content of the manuscript.

### Copyright

© The Author(s) 2026.
