# Supplementary material for: Optimized enrichment of circulating extracellular vesicles from whole blood samples using PROSPR
Source: Extracell Vesicles Circ Nucl Acids. 2026 Jul 7;7(3):1084–107. doi: 10.20517/evcna.2026.18 (PMC13673457; doi:10.20517/evcna.2026.18)
Supplement: Supplementary file 1 [file evcna-7-3-1084-SupplementaryMaterials.zip › evcna7018-SupplementaryMaterials.pdf]

Supplementary Materials

Optimized enrichment of circulating extracellular vesicles from whole blood samples using PROSPR

Jose Antonio Sánchez Milán[1,2], María Mulet[1,2], Maria Font-Alberich[1,2], Magdalena Torres[1,2], Yormaris Castillo[1,2], Silvia Pico[3], Oriol Yuguero[4,5], Gerard Piñol-Ripoll[6], Filip Bellon[7,8], Montserrat Gea-Sánchez[7,8,9], Siu Kwan Sze[10], Raj N. Kalaria[11], Xavier Gallart-Palau[12], Aida Serra[1,2]

[1]Biomedical Research Institute of Lleida Dr. Pifarré Foundation (IRBLleida), +Pec Proteomics Research Group (+PPRG), Neuroscience Area, Lleida 25198, Spain.

[2]Department of Medical Basic Sciences, University of Lleida (UdL), Lleida 25198, Spain.

[3]Laboratory Medicine Department, University Hospital Arnau de Vilanova, Lleida 25198, Spain.

[4]ERLab Research Group, Biomedical Research Institute of Lleida Dr. Pifarré Foundation (IRBLleida), Lleida 25198, Spain.

[5]Department of Medicine, Faculty of Medicine, University of Lleida (UdL), Lleida 25198, Spain.

[6]Clinical Neuroscience Research, Unitat Trastorns Cognitius, Biomedical Research Institute of Lleida Dr. Pifarré Foundation (IRBLleida), Santa Maria University Hospital, Lleida 25198, Spain.

[7]Healthcare Research Group (GRECS), Biomedical Research Institute of Lleida Dr. Pifarré Foundation (IRBLleida), Lleida 25198, Spain.

[8]Grup d'Estudis Salut, Societat, Educació i Cultura de les Cures (GESEC), Departament d'Infermeria i Fisiotèrapia, Universitat de Lleida, Lleida 25198, Spain.

[9]CIBER Fragilidad y Envejecimiento Saludable (CIBERFES), Instituto de Salud Carlos III, Lleida 25198, Spain.

[10]Department of Health Sciences, Faculty of Applied Health Sciences, Brock University, St. Catharines L2S 3A1, Canada.

[11]Translational and Clinical Research Institute, Campus for Ageing and Vitality, Newcastle University, Newcastle upon Tyne NE4 6BE, UK.

[12]Biomedical Research Institute of Lleida Dr. Pifarré Foundation (IRBLleida), +Pec Proteomics Research Group (+PPRG), Neuroscience Area, University Hospital Arnau de Vilanova (HUAV), Lleida 25198, Spain.

Correspondence to: Dr. Xavier Gallart-Palau, Biomedical Research Institute of Lleida Dr. Pifarré Foundation (IRBLleida), +Pec Proteomics Research Group (+PPRG), Neuroscience Area, University Hospital Arnau de Vilanova (HUAV), Lleida 25198, Spain. E-mail: xgallart@irblleida.cat

Supplementary Table 7. List of proteins associated to biological pathways linked to exclusive proteins identified only in blood EVs enriched by PROSPR

Pathway	Pathway ID	Protein accession	Description
VEGFA-VEGFR2 signaling	WP3888_126337	Q00610	clathrin heavy chain
VEGFA-VEGFR2 signaling	WP3888_126337	P02671	fibrinogen alpha chain
VEGFA-VEGFR2 signaling	WP3888_126337	P31948	stress induced phosphoprotein 1
VEGFA-VEGFR2 signaling	WP3888_126337	P00338	lactate dehydrogenase A
VEGFA-VEGFR2 signaling	WP3888_126337	Q5TZZ9	annexin A1
VEGFA-VEGFR2 signaling	WP3888_126337	Q6IBT3	chaperonin containing TCP1 subunit 7
VEGFA-VEGFR2 signaling	WP3888_126337	P52209	phosphogluconate dehydrogenase
VEGFA-VEGFR2 signaling	WP3888_126337	P61224	RAP1B, member of RAS oncogene family
VEGFA-VEGFR2 signaling	WP3888_126337	Q5SRT3	chloride intracellular channel 1
VEGFA-VEGFR2 signaling	WP3888_126337	O00231	proteasome 26S subunit, non-ATPase 11
VEGFA-VEGFR2 signaling	WP3888_126337	A4D2P0	Rac family small GTPase 1
VEGFA-VEGFR2 signaling	WP3888_126337	B3KUN1	protein phosphatase 2 catalytic subunit alpha
VEGFA-VEGFR2 signaling	WP3888_126337	P60953	cell division cycle 42
VEGFA-VEGFR2 signaling	WP3888_126337	Q9HB00	desmocollin 1
VEGFA-VEGFR2	WP3888_126337	Q59EF6	calpain 2

signaling			
VEGFA-VEGFR2 signaling	WP3888_126337	B4DMN1	RAB11A, member RAS oncogene family
Complement system	WP2806_127174	B4E1E1	complement component 4 binding protein alpha
Complement system	WP2806_127174	P02671	fibrinogen alpha chain
Complement system	WP2806_127174	P02671	fibrinogen alpha chain
Complement system	WP2806_127174	A8K9A9	kallikrein B1
Complement system	WP2806_127174	P08246	elastase, neutrophil expressed
Alzheimer's disease	WP5124_128389	Q13564	NEDD8 activating enzyme E1 subunit 1
Alzheimer's disease	WP5124_128389	B4DJ51	calmodulin 1
Alzheimer's disease	WP5124_128389	A8JZY9	tubulin alpha 4a
Alzheimer's disease	WP5124_128389	B4DJ51	calmodulin 1
Alzheimer's disease	WP5124_128389	B4DJ51	calmodulin 1
Alzheimer's disease	WP5124_128389	Q9NZT1	calmodulin like 5
Alzheimer's disease	WP5124_128389	B7Z478	proteasome 20S subunit beta 2
Alzheimer's disease	WP5124_128389	Q6IB71	proteasome 20S subunit alpha 3
Alzheimer's disease	WP5124_128389	Q05DH1	proteasome 20S subunit alpha 7
Alzheimer's disease	WP5124_128389	P07384	calpain 1
Alzheimer's disease	WP5124_128389	P25786	proteasome 20S subunit alpha 1
Alzheimer's disease	WP5124_128389	Q59EF6	calpain 2
Alzheimer's disease	WP5124_128389	Q4W5L2	synuclein alpha
Alzheimer's disease	WP5124_128389	P28074	proteasome 20S subunit beta 5
Alzheimer's disease and miRNA effects	WP2059_128085	A8JZY9	tubulin alpha 4a
Alzheimer's disease	WP2059_128085	Q13564	NEDD8 activating enzyme

and miRNA effects			E1 subunit 1
Alzheimer's disease and miRNA effects	WP2059_128085	B4DJ51	calmodulin 1
Alzheimer's disease and miRNA effects	WP2059_128085	B4DJ51	calmodulin 1
Alzheimer's disease and miRNA effects	WP2059_128085	B4DJ51	calmodulin 1
Alzheimer's disease and miRNA effects	WP2059_128085	Q9NZT1	calmodulin like 5
Alzheimer's disease and miRNA effects	WP2059_128085	B7Z478	proteasome 20S subunit beta 2
Alzheimer's disease and miRNA effects	WP2059_128085	Q6IB71	proteasome 20S subunit alpha 3
Alzheimer's disease and miRNA effects	WP2059_128085	Q05DH1	proteasome 20S subunit alpha 7
Alzheimer's disease and miRNA effects	WP2059_128085	P25786	proteasome 20S subunit alpha 1
Alzheimer's disease and miRNA effects	WP2059_128085	P28074	proteasome 20S subunit beta 5
Alzheimer's disease and miRNA effects	WP2059_128085	P07384	calpain 1
Alzheimer's disease and miRNA effects	WP2059_128085	Q59EF6	calpain 2
Alzheimer's disease and miRNA effects	WP2059_128085	Q4W5L2	synuclein alpha
Complement and coagulation cascades	WP558_126246	Q00610	clathrin heavy chain
Complement and coagulation cascades	WP558_126246	P10909	clusterin
Complement and coagulation cascades	WP558_126246	A8K9A9	kallikrein B1
Complement and coagulation cascades	WP558_126246	B4DPP8	kininogen 1

Dengue-2 interactions with complement and coagulation cascades	WP3896_129065	Q00610	clathrin heavy chain
Dengue-2 interactions with complement and coagulation cascades	WP3896_129065	P10909	clusterin
Dengue-2 interactions with complement and coagulation cascades	WP3896_129065	A8K9A9	kallikrein B1
Dengue-2 interactions with complement and coagulation cascades	WP3896_129065	B4DPP8	kininogen 1
Metabolic epileptic disorders	WP5355_129474	P08237	phosphofructokinase, muscle
Metabolic epileptic disorders	WP5355_129474	P05089	arginase 1
Metabolic epileptic disorders	WP5355_129474	P60174	triosephosphate isomerase 1
Metabolic epileptic disorders	WP5355_129474	P30613	pyruvate kinase L/R
Metabolic epileptic disorders	WP5355_129474	P00338	lactate dehydrogenase A
Metabolic epileptic disorders	WP5355_129474	B4DE36	glucose-6-phosphate isomerase
Metabolic epileptic disorders	WP5355_129474	P11166	solute carrier family 2 member 1
Metabolic epileptic disorders	WP5355_129474	B7Z1Y2	aldolase, fructose-bisphosphate C
Metabolic epileptic disorders	WP5355_129474	P17858	phosphofructokinase, liver type
Metabolic epileptic disorders	WP5355_129474	P17174	glutamic-oxaloacetic transaminase 1
Selenium	WP15_125000	C0JYY2	adenosylhomocysteinase

micronutrient network			
Selenium micronutrient network	WP15_125000	P02671	fibrinogen alpha chain
Selenium micronutrient network	WP15_125000	B2R5G8	serum amyloid A4, constitutive
Selenium micronutrient network	WP15_125000	P05164	myeloperoxidase
Selenium micronutrient network	WP15_125000	P04040	catalase
Clear cell renal cell carcinoma pathways	WP4018_127315	B4DE36	glucose-6-phosphate isomerase
Clear cell renal cell carcinoma pathways	WP4018_127315	P17858	phosphofructokinase, liver type
Clear cell renal cell carcinoma pathways	WP4018_127315	P08237	phosphofructokinase, muscle
Clear cell renal cell carcinoma pathways	WP4018_127315	B7Z1Y2	aldolase, fructose-bisphosphate C
Clear cell renal cell carcinoma pathways	WP4018_127315	P60174	triosephosphate isomerase 1
Clear cell renal cell carcinoma pathways	WP4018_127315	P30613	pyruvate kinase L/R
Clear cell renal cell carcinoma pathways	WP4018_127315	P00338	lactate dehydrogenase A
Clear cell renal cell carcinoma pathways	WP4018_127315	P11166	solute carrier family 2 member 1
Clear cell renal cell carcinoma pathways	WP4018_127315	P53396	ATP citrate lyase
Clear cell renal cell	WP4018_127315	Q96G03	phosphoglucomutase 2

carcinoma pathways			
Network map of SARS-CoV-2 signaling	WP5115_129746	B2R8I2	histidine rich glycoprotein
Network map of SARS-CoV-2 signaling	WP5115_129746	P06396	gelsolin
Network map of SARS-CoV-2 signaling	WP5115_129746	Q1KLZ0	actin beta
Network map of SARS-CoV-2 signaling	WP5115_129746	P05090	apolipoprotein D
Network map of SARS-CoV-2 signaling	WP5115_129746	A4D2P0	Rac family small GTPase 1
Network map of SARS-CoV-2 signaling	WP5115_129746	P02671	fibrinogen alpha chain
Glycolysis and gluconeogenesis	WP534_128076	P60174	triosephosphate isomerase 1
Glycolysis and gluconeogenesis	WP534_128076	P08237	phosphofructokinase, muscle
Glycolysis and gluconeogenesis	WP534_128076	P17858	phosphofructokinase, liver type
Glycolysis and gluconeogenesis	WP534_128076	B4DE36	glucose-6-phosphate isomerase
Glycolysis and gluconeogenesis	WP534_128076	P30613	pyruvate kinase L/R
Glycolysis and gluconeogenesis	WP534_128076	B7Z1Y2	aldolase, fructose-bisphosphate C
Glycolysis and gluconeogenesis	WP534_128076	P17174	glutamic-oxaloacetic transaminase 1

Glycolysis and gluconeogenesis	WP534_128076	P00338	lactate dehydrogenase A
Glycolysis and gluconeogenesis	WP534_128076	P11166	solute carrier family 2 member 1
Vitamin B12 metabolism	WP1533_130784	B2R5G8	serum amyloid A4, constitutive
Vitamin B12 metabolism	WP1533_130784	P02671	fibrinogen alpha chain
Vitamin B12 metabolism	WP1533_130784	C0JYY2	apolipoprotein B-100
Vitamin B12 metabolism	WP1533_130784	P05164	myeloperoxidase
16p11.2 proximal deletion syndrome	WP4949_125693	P30153	protein phosphatase 2 scaffold subunit Aalpha
16p11.2 proximal deletion syndrome	WP4949_125693	P50990	chaperonin containing TCP1 subunit 8
16p11.2 proximal deletion syndrome	WP4949_125693	Q6IBT3	chaperonin containing TCP1 subunit 7
16p11.2 proximal deletion syndrome	WP4949_125693	P50991	chaperonin containing TCP1 subunit 4
16p11.2 proximal deletion syndrome	WP4949_125693	B4DX08	chaperonin containing TCP1 subunit 5
16p11.2 proximal deletion syndrome	WP4949_125693	P49368	chaperonin containing TCP1 subunit 3
16p11.2 proximal deletion syndrome	WP4949_125693	B3KUN1	protein phosphatase 2 catalytic subunit alpha
Folate metabolism	WP176_121696	B2R5G8	serum amyloid A4, constitutive
Folate metabolism	WP176_121696	C0JYY2	apolipoprotein B-100
Folate metabolism	WP176_121696	P02671	fibrinogen alpha chain
Folate metabolism	WP176_121696	P05164	myeloperoxidase
Folate metabolism	WP176_121696	P04040	catalase
Proteasome	WP183_128354	Q9UFQ0	ubiquitin B

degradation			
Proteasome degradation	WP183_128354	Q08AJ9	H2A clustered histone 8
Proteasome degradation	WP183_128354	O00231	proteasome 26S subunit, non-ATPase 11
Proteasome degradation	WP183_128354	P22314	ubiquitin like modifier activating enzyme 1
Proteasome degradation	WP183_128354	B7Z478	proteasome 20S subunit beta 2
Proteasome degradation	WP183_128354	Q05DH1	proteasome 20S subunit alpha 7
Proteasome degradation	WP183_128354	P25786	proteasome 20S subunit alpha 1
Proteasome degradation	WP183_128354	Q9UL46	proteasome activator subunit 2
Proteasome degradation	WP183_128354	Q6IB71	proteasome 20S subunit alpha 3
Proteasome degradation	WP183_128354	P28074	proteasome 20S subunit beta 5
Ras signaling	WP4223_127495	B4DJ51	calmodulin 1
Ras signaling	WP4223_127495	B4DJ51	calmodulin 1
Ras signaling	WP4223_127495	Q9NZT1	calmodulin like 5
Ras signaling	WP4223_127495	A4D2P0	Rac family small GTPase 1
Ras signaling	WP4223_127495	Q6FI54	RAB5B, member RAS oncogene family
Ras signaling	WP4223_127495	P51148	RAB5C, member RAS oncogene family
Ras signaling	WP4223_127495	P0C869	JMJD7-PLA2G4B readthrough
Ras signaling	WP4223_127495	P60953	cell division cycle 42
Ras signaling	WP4223_127495	P61224	RAP1B, member of RAS oncogene family

Ras signaling	WP4223_127495	B4DJ51	calmodulin 1
Nuclear receptors meta-pathway	WP2882_126680	B4DZK8	stomatin
Nuclear receptors meta-pathway	WP2882_126680	P11413	glucose-6-phosphate dehydrogenase
Nuclear receptors meta-pathway	WP2882_126680	P52209	phosphogluconate dehydrogenase
Nuclear receptors meta-pathway	WP2882_126680	P11166	solute carrier family 2 member 1
Nuclear receptors meta-pathway	WP2882_126680	P48507	glutamate-cysteine ligase modifier subunit
Nuclear receptors meta-pathway	WP2882_126680	Q14TF0	glutamate-cysteine ligase catalytic subunit
Nuclear receptors meta-pathway	WP2882_126680	P30043	biliverdin reductase B
Nuclear receptors meta-pathway	WP2882_126680	A3KPE2	apolipoprotein C3
Metabolic reprogramming in colon cancer	WP4290_118611	P00338	lactate dehydrogenase A
Metabolic reprogramming in colon cancer	WP4290_118611	B4DE36	glucose-6-phosphate isomerase
Metabolic reprogramming in colon cancer	WP4290_118611	P49247	ribose 5-phosphate isomerase A
Metabolic reprogramming in colon cancer	WP4290_118611	P17858	phosphofructokinase, liver type
Metabolic reprogramming in colon cancer	WP4290_118611	P53396	ATP citrate lyase
Metabolic	WP4290_118611	P52209	phosphogluconate

reprogramming in colon cancer			dehydrogenase
Metabolic reprogramming in colon cancer	WP4290_118611	P11413	glucose-6-phosphate dehydrogenase
Metabolic reprogramming in colon cancer	WP4290_118611	P11166	solute carrier family 2 member 1
Cancer pathways	WP5434_129459	Q9NZT1	calmodulin like 5
Cancer pathways	WP5434_129459	P11166	solute carrier family 2 member 1
Cancer pathways	WP5434_129459	B4DPP8	kininogen 1
Cancer pathways	WP5434_129459	A4D2P0	Rac family small GTPase 1
Cancer pathways	WP5434_129459	P78417	glutathione S-transferase omega 1
Cancer pathways	WP5434_129459	P63208	S-phase kinase associated protein 1

Supplementary Table 8. List of proteins associated to biological pathways linked to exclusive proteins identified only in plasma EVs enriched by PROSPR

Pathway	Pathway ID	UNIPROT	Description
VEGFA-VEGFR2 signaling	WP3888_126337	P42566	epidermal growth factor receptor pathway substrate 15
VEGFA-VEGFR2 signaling	WP3888_126337	P00367	glutamate dehydrogenase 1
VEGFA-VEGFR2 signaling	WP3888_126337	P42345	mechanistic target of rapamycin kinase
VEGFA-VEGFR2 signaling	WP3888_126337	B3KR49	mitogen-activated protein kinase
VEGFA-VEGFR2 signaling	WP3888_126337	A8MYV2	LUC7 like
VEGFA-VEGFR2 signaling	WP3888_126337	A2A3R7	small ribosomal subunit protein eS6
VEGFA-VEGFR2 signaling	WP3888_126337	B1PS43	myosin heavy chain 11
VEGFA-VEGFR2 signaling	WP3888_126337	Q02156	protein kinase C epsilon
VEGFA-VEGFR2 signaling	WP3888_126337	P04792	heat shock protein family B (small) member 1
VEGFA-VEGFR2 signaling	WP3888_126337	P55036	proteasome 26S subunit ubiquitin receptor, non-ATPase 4
VEGFA-VEGFR2 signaling	WP3888_126337	Q15293	reticulocalbin 1
VEGFA-VEGFR2 signaling	WP3888_126337	Q5SZK4	WASP family member 1
VEGFA-VEGFR2 signaling	WP3888_126337	P05387	ribosomal protein lateral stalk subunit P2
VEGFA-VEGFR2 signaling	WP3888_126337	Q5T5C7	seryl-tRNA synthetase 1

VEGFA-VEGFR2 signaling	WP3888_126337	C9J0K5	NCK adaptor protein 1
VEGFA-VEGFR2 signaling	WP3888_126337	Q9H8L6	multimerin 2
VEGFA-VEGFR2 signaling	WP3888_126337	B1ALY0	PALM2 and AKAP2 fusion
VEGFA-VEGFR2 signaling	WP3888_126337	P36543	ATPase H+ transporting V1 subunit E1
VEGFA-VEGFR2 signaling	WP3888_126337	P04075	aldolase, fructose-bisphosphate A
VEGFA-VEGFR2 signaling	WP3888_126337	P62328	thymosin beta 4 X-linked
VEGFA-VEGFR2 signaling	WP3888_126337	P02675	fibrinogen beta chain
VEGFA-VEGFR2 signaling	WP3888_126337	C9JAK5	ADP ribosylation factor 4
VEGFA-VEGFR2 signaling	WP3888_126337	Q5T3N1	annexin
VEGFA-VEGFR2 signaling	WP3888_126337	B1AK85	capping actin protein of muscle Z-line subunit beta
VEGFA-VEGFR2 signaling	WP3888_126337	Q9BPX5	actin related protein 2/3 complex subunit 5 like
VEGFA-VEGFR2 signaling	WP3888_126337	Q9Y5S2	CDC42 binding protein kinase beta
VEGFA-VEGFR2 signaling	WP3888_126337	A0AVL1	ADAM metallopeptidase domain 9
VEGFA-VEGFR2 signaling	WP3888_126337	Q5VU77	Ubiquitin associated protein 2 like
VEGFA-VEGFR2 signaling	WP3888_126337	P46940	IQ motif containing GTPase activating protein 1
VEGFA-VEGFR2 signaling	WP3888_126337	Q08209	protein phosphatase 3 catalytic subunit alpha
VEGFA-VEGFR2	WP3888_126337	Q9UDY4	DnaJ heat shock protein

signaling			family (Hsp40) member B4
VEGFA-VEGFR2 signaling	WP3888_126337	Q13464	Rho associated coiled-coil containing protein kinase 1
VEGFA-VEGFR2 signaling	WP3888_126337	P02042	hemoglobin subunit delta
VEGFA-VEGFR2 signaling	WP3888_126337	P04406	glyceraldehyde-3-phosphate dehydrogenase
Complement system	WP2806_127174	Q5VVP7	C-reactive protein
Complement system	WP2806_127174	P10643	complement C7
Complement system	WP2806_127174	P04003	complement component 4 binding protein alpha
Complement system	WP2806_127174	P02748	complement C9
Complement system	WP2806_127174	P27918	complement factor properdin
Complement system	WP2806_127174	P07357	complement C8 alpha chain
Complement system	WP2806_127174	P02675	fibrinogen beta chain
Complement system	WP2806_127174	P08754	G protein subunit alpha i3
Complement system	WP2806_127174	P02647	apolipoprotein A1
Complement system	WP2806_127174	O15230	laminin subunit alpha 5
Complement system	WP2806_127174	B4E1Z4	Complement C2
Complement system	WP2806_127174	P02675	fibrinogen beta chain
Alzheimer’s disease	WP5124_128389	B3KR49	mitogen-activated protein kinase
Alzheimer’s disease	WP5124_128389	B1AM21	G protein subunit alpha q
Alzheimer’s disease	WP5124_128389	P01111	NRAS proto-oncogene, GTPase
Alzheimer’s disease	WP5124_128389	P33176	kinesin family member 5B
Alzheimer’s disease	WP5124_128389	Q5H928	Hydroxysteroid (17-beta) dehydrogenase 10
Alzheimer’s disease	WP5124_128389	P68363	tubulin alpha 1b
Alzheimer’s disease	WP5124_128389	P55036	proteasome 26S subunit ubiquitin receptor, non-ATPase 4

Alzheimer’s disease	WP5124_128389	B3KR49	mitogen-activated protein kinase
Alzheimer’s disease	WP5124_128389	O43242	proteasome 26S subunit, non-ATPase 3
Alzheimer’s disease	WP5124_128389	Q08209	protein phosphatase 3 catalytic subunit alpha
Alzheimer’s disease	WP5124_128389	Q5F2G0	Protein phosphatase 3 catalytic subunit beta
Alzheimer’s disease	WP5124_128389	P42345	mechanistic target of rapamycin kinase
Alzheimer’s disease	WP5124_128389	P04350	tubulin beta 4A class IVa
Alzheimer’s disease	WP5124_128389	P05141	solute carrier family 25 member 5
Alzheimer’s disease and miRNA effects	WP2059_128085	P01111	NRAS proto-oncogene, GTPase
Alzheimer’s disease and miRNA effects	WP2059_128085	P33176	kinesin family member 5B
Alzheimer’s disease and miRNA effects	WP2059_128085	B1AM21	G protein subunit alpha q
Alzheimer’s disease and miRNA effects	WP2059_128085	P68363	tubulin alpha 1b
Alzheimer’s disease and miRNA effects	WP2059_128085	P55036	proteasome 26S subunit ubiquitin receptor, non-ATPase 4
Alzheimer’s disease and miRNA effects	WP2059_128085	Q5H928	Hydroxysteroid (17-beta) dehydrogenase 10
Alzheimer’s disease and miRNA effects	WP2059_128085	O43242	proteasome 26S subunit, non-ATPase 3
Alzheimer’s disease and miRNA effects	WP2059_128085	B3KR49	mitogen-activated protein kinase
Alzheimer’s disease and miRNA effects	WP2059_128085	P42345	mechanistic target of rapamycin kinase
Alzheimer’s disease and	WP2059_128085	P04350	tubulin beta 4A class IVa

miRNA effects			
Alzheimer's disease and miRNA effects	WP2059_128085	P05141	solute carrier family 25 member 5
Alzheimer's disease and miRNA effects	WP2059_128085	Q08209	protein phosphatase 3 catalytic subunit alpha
Alzheimer's disease and miRNA effects	WP2059_128085	P68371	tubulin beta 4B class IVb
Alzheimer's disease and miRNA effects	WP2059_128085	P12236	solute carrier family 25 member 6
Alzheimer's disease and miRNA effects	WP2059_128085	Q5F2G0	Protein phosphatase 3 catalytic subunit beta
Alzheimer's disease and miRNA effects	WP2059_128085	P07437	tubulin beta class I
Alzheimer's disease and miRNA effects	WP2059_128085	P21796	voltage dependent anion channel 1
Alzheimer's disease and miRNA effects	WP2059_128085	Q13885	tubulin beta 2A class IIa
Alzheimer's disease and miRNA effects	WP2059_128085	Q9H4B7	tubulin beta 1 class VI
Alzheimer's disease and miRNA effects	WP2059_128085	P04406	glyceraldehyde-3-phosphate dehydrogenase
Alzheimer's disease and miRNA effects	WP2059_128085	B3KR49	mitogen-activated protein kinase
Complement and coagulation cascades	WP558_126246	P01009	serpin family A member 1
Complement and coagulation cascades	WP558_126246	P02675	fibrinogen beta chain
Complement and coagulation cascades	WP558_126246	P05160	coagulation factor XIII B chain
Complement and coagulation cascades	WP558_126246	P02748	complement C9
Complement and coagulation cascades	WP558_126246	P02747	complement C1q C chain

Complement and coagulation cascades	WP558_126246	B4E1Z4	Complement C2
Complement and coagulation cascades	WP558_126246	P10643	complement C7
Dengue-2 interactions with complement and coagulation cascades	WP3896_129065	P01009	serpin family A member 1
Dengue-2 interactions with complement and coagulation cascades	WP3896_129065	P02675	fibrinogen beta chain
Dengue-2 interactions with complement and coagulation cascades	WP3896_129065	P05160	coagulation factor XIII B chain
Dengue-2 interactions with complement and coagulation cascades	WP3896_129065	P02748	complement C9
Dengue-2 interactions with complement and coagulation cascades	WP3896_129065	P02747	complement C1q C chain
Dengue-2 interactions with complement and coagulation cascades	WP3896_129065	B4E1Z4	B4E1Z4
Dengue-2 interactions with complement and coagulation cascades	WP3896_129065	P10643	complement C7
Metabolic epileptic disorders	WP5355_129474	P06733	enolase 1
Metabolic epileptic disorders	WP5355_129474	P23378	glycine decarboxylase
Metabolic epileptic disorders	WP5355_129474	P09104	enolase 2
Metabolic epileptic disorders	WP5355_129474	P04075	aldolase, fructose-bisphosphate A

Metabolic epileptic disorders	WP5355_129474	P09972	aldolase, fructose-bisphosphate C
Metabolic epileptic disorders	WP5355_129474	P04406	glyceraldehyde-3-phosphate dehydrogenase
Metabolic epileptic disorders	WP5355_129474	P11169	solute carrier family 2 member 3
Metabolic epileptic disorders	WP5355_129474	P24752	acetyl-CoA acetyltransferase 1
Metabolic epileptic disorders	WP5355_129474	P00367	glutamate dehydrogenase 1
Metabolic epileptic disorders	WP5355_129474	P23378	glycine decarboxylase
Metabolic epileptic disorders	WP5355_129474	P51687	sulfite oxidase
Metabolic epileptic disorders	WP5355_129474	P30084	enoyl-CoA hydratase, short chain 1
Metabolic epileptic disorders	WP5355_129474	P11498	pyruvate carboxylase
Metabolic epileptic disorders	WP5355_129474	P00505	glutamic-oxaloacetic transaminase 2
Metabolic epileptic disorders	WP5355_129474	P05141	solute carrier family 25 member 5
Metabolic epileptic disorders	WP5355_129474	P40926	malate dehydrogenase 2
Metabolic epileptic disorders	WP5355_129474	O43175	phosphoglycerate dehydrogenase
Selenium micronutrient network	WP15_125000	P02647	apolipoprotein A1
Selenium micronutrient network	WP15_125000	P04114	apolipoprotein B
Selenium micronutrient network	WP15_125000	P02675	fibrinogen beta chain
Selenium micronutrient	WP15_125000	Q06830	peroxiredoxin 1

network			
Selenium micronutrient network	WP15_125000	Q5VVP7	C-reactive protein
Selenium micronutrient network	WP15_125000	P69905	hemoglobin subunit alpha 1
Selenium micronutrient network	WP15_125000	P00441	superoxide dismutase 1
Clear cell renal cell carcinoma pathways	WP4018_127315	P09104	enolase 2
Clear cell renal cell carcinoma pathways	WP4018_127315	O43175	phosphoglycerate dehydrogenase
Clear cell renal cell carcinoma pathways	WP4018_127315	P42345	mechanistic target of rapamycin kinase
Clear cell renal cell carcinoma pathways	WP4018_127315	P49327	fatty acid synthase
Clear cell renal cell carcinoma pathways	WP4018_127315	P04075	aldolase, fructose-bisphosphate A
Clear cell renal cell carcinoma pathways	WP4018_127315	P09972	aldolase, fructose-bisphosphate C
Clear cell renal cell carcinoma pathways	WP4018_127315	P04406	glyceraldehyde-3-phosphate dehydrogenase
Clear cell renal cell carcinoma pathways	WP4018_127315	Q14012	calcium/calmodulin dependent protein kinase I
Clear cell renal cell carcinoma pathways	WP4018_127315	P06733	enolase 1
Network map of SARS-CoV-2 signaling	WP5115_129746	Q5VVP7	C-reactive protein
Network map of SARS-CoV-2 signaling	WP5115_129746	P27918	complement factor properdin
Network map of SARS-CoV-2 signaling	WP5115_129746	P02675	fibrinogen beta chain
Network map of SARS-CoV-2 signaling	WP5115_129746	P23458	Janus kinase 1

Network map of SARS-CoV-2 signaling	WP5115_129746	P18428	lipopolysaccharide binding protein
Network map of SARS-CoV-2 signaling	WP5115_129746	P07357	complement C8 alpha chain
Network map of SARS-CoV-2 signaling	WP5115_129746	A2A3R7	Small ribosomal subunit protein eS6
Network map of SARS-CoV-2 signaling	WP5115_129746	P04217	alpha-1-B glycoprotein
Network map of SARS-CoV-2 signaling	WP5115_129746	Q5VVP7	C-reactive protein
Network map of SARS-CoV-2 signaling	WP5115_129746	P02042	hemoglobin subunit delta
Network map of SARS-CoV-2 signaling	WP5115_129746	O95786	RNA sensor RIG-I
Network map of SARS-CoV-2 signaling	WP5115_129746	P01019	angiotensinogen
Network map of SARS-CoV-2 signaling	WP5115_129746	P02675	fibrinogen beta chain
Network map of SARS-CoV-2 signaling	WP5115_129746	P04196	histidine rich glycoprotein
Network map of SARS-CoV-2 signaling	WP5115_129746	Q5VVP7	C-reactive protein
Network map of SARS-CoV-2 signaling	WP5115_129746	P18428	lipopolysaccharide binding protein
Network map of SARS-CoV-2 signaling	WP5115_129746	P02749	apolipoprotein H
Network map of SARS-CoV-2 signaling	WP5115_129746	P02750	leucine rich alpha-2-glycoprotein 1
Network map of SARS-CoV-2 signaling	WP5115_129746	B4E1Z4	Complement C2
Network map of SARS-CoV-2 signaling	WP5115_129746	P42345	mechanistic target of rapamycin kinase
Network map of SARS-	WP5115_129746	P02647	apolipoprotein A1

CoV-2 signaling			
Network map of SARS-CoV-2 signaling	WP5115_129746	P15814	immunoglobulin lambda like polypeptide 1
Glycolysis and gluconeogenesis	WP534_128076	P11498	pyruvate carboxylase
Glycolysis and gluconeogenesis	WP534_128076	P00505	glutamic-oxaloacetic transaminase 2
Glycolysis and gluconeogenesis	WP534_128076	P04075	aldolase, fructose-bisphosphate A
Glycolysis and gluconeogenesis	WP534_128076	P06733	enolase 1
Glycolysis and gluconeogenesis	WP534_128076	P09972	aldolase, fructose-bisphosphate C
Glycolysis and gluconeogenesis	WP534_128076	P40926	malate dehydrogenase 2
Glycolysis and gluconeogenesis	WP534_128076	P11169	solute carrier family 2 member 3
Glycolysis and gluconeogenesis	WP534_128076	P04406	glyceraldehyde-3-phosphate dehydrogenase
Glycolysis and gluconeogenesis	WP534_128076	P09104	enolase 2
Vitamin B12 metabolism	WP1533_130784	P02647	apolipoprotein A1
Vitamin B12 metabolism	WP1533_130784	P69905	hemoglobin subunit alpha 1
Vitamin B12 metabolism	WP1533_130784	Q5VVP7	C-reactive protein
Vitamin B12 metabolism	WP1533_130784	P00441	superoxide dismutase 1
Vitamin B12 metabolism	WP1533_130784	P02675	fibrinogen beta chain
Vitamin B12 metabolism	WP1533_130784	P22033	methylmalonyl-CoA mutase

Vitamin B12 metabolism	WP1533_130784	P04114	apolipoprotein B
16p11.2 proximal deletion syndrome	WP4949_125693	B3KR49	mitogen-activated protein kinase
16p11.2 proximal deletion syndrome	WP4949_125693	Q16204	coiled-coil domain containing 6
16p11.2 proximal deletion syndrome	WP4949_125693	B7ZAR1	chaperonin containing TCP1 subunit 5
16p11.2 proximal deletion syndrome	WP4949_125693	P04075	aldolase, fructose-bisphosphate A
Folate metabolism	WP176_121696	P69905	hemoglobin subunit alpha 1
Folate metabolism	WP176_121696	P00441	superoxide dismutase 1
Folate metabolism	WP176_121696	P04114	apolipoprotein B
Folate metabolism	WP176_121696	P02675	fibrinogen beta chain
Folate metabolism	WP176_121696	P02647	apolipoprotein A1
Folate metabolism	WP176_121696	Q5VVP7	C-reactive protein
Proteasome degradation	WP183_128354	O43242	proteasome 26S subunit, non-ATPase 3
Proteasome degradation	WP183_128354	P55036	proteasome 26S subunit ubiquitin receptor, non-ATPase 4
Proteasome degradation	WP183_128354	Q2L6I5	Major histocompatibility complex, class I, E
Proteasome degradation	WP183_128354	Q5SRN5	Major histocompatibility complex, class I, A
Proteasome degradation	WP183_128354	B3KQ25	proteasome activator subunit 3
Ras signaling	WP4223_127495	O14807	muscle RAS oncogene homolog
Ras signaling	WP4223_127495	B3KR49	mitogen-activated protein kinase
Ras signaling	WP4223_127495	P01111	NRAS proto-oncogene, GTPase

Ras signaling	WP4223_127495	Q5TCY1	tau tubulin kinase 1
Ras signaling	WP4223_127495	Q9HAV0	G protein subunit beta 4
Nuclear receptors meta-pathway	WP2882_126680	P55058	phospholipid transfer protein
Nuclear receptors meta-pathway	WP2882_126680	P23786	carnitine palmitoyltransferase 2
Nuclear receptors meta-pathway	WP2882_126680	P02647	apolipoprotein A1
Nuclear receptors meta-pathway	WP2882_126680	Q5T4U5	acyl-CoA dehydrogenase medium chain
Nuclear receptors meta-pathway	WP2882_126680	Q9ULF5	solute carrier family 39 member 10
Nuclear receptors meta-pathway	WP2882_126680	P02689	peripheral myelin protein 2
Nuclear receptors meta-pathway	WP2882_126680	O95810	caveolae associated protein 2
Nuclear receptors meta-pathway	WP2882_126680	P01009	serpin family A member 1
Nuclear receptors meta-pathway	WP2882_126680	O00767	stearoyl-CoA desaturase
Nuclear receptors meta-pathway	WP2882_126680	O75828	carbonyl reductase 3
Nuclear receptors meta-pathway	WP2882_126680	Q5T948	rotein phosphatase 2 phosphatase activator
Nuclear receptors meta-pathway	WP2882_126680	Q06830	peroxiredoxin 1
Nuclear receptors meta-pathway	WP2882_126680	P27105	stomatin
Nuclear receptors meta-pathway	WP2882_126680	P50453	serpin family B member 9
Nuclear receptors meta-pathway	WP2882_126680	P16152	carbonyl reductase 1
Nuclear receptors meta-	WP2882_126680	Q5T8R1	glutathione S-transferase

pathway			mu 5
Nuclear receptors meta-pathway	WP2882_126680	P08238	heat shock protein 90 alpha family class B member 1
Nuclear receptors meta-pathway	WP2882_126680	P11169	solute carrier family 2 member 3
Nuclear receptors meta-pathway	WP2882_126680	Q13451	FKBP prolyl isomerase 5
Nuclear receptors meta-pathway	WP2882_126680	C9J5P8	Solute carrier family 6 member 1
Nuclear receptors meta-pathway	WP2882_126680	Q96QE2	solute carrier family 2 member 13
Nuclear receptors meta-pathway	WP2882_126680	P49327	fatty acid synthase
Nuclear receptors meta-pathway	WP2882_126680	C9JDE9	acetyl-CoA acyltransferase 1
Nuclear receptors meta-pathway	WP2882_126680	B0YIW2	apolipoprotein C3
Metabolic reprogramming in colon cancer	WP4290_118611	P06733	enolase 1
Metabolic reprogramming in colon cancer	WP4290_118611	P00505	glutamic-oxaloacetic transaminase 2
Metabolic reprogramming in colon cancer	WP4290_118611	P40926	malate dehydrogenase 2
Metabolic reprogramming in colon cancer	WP4290_118611	P49327	fatty acid synthase
Metabolic reprogramming in colon cancer	WP4290_118611	P04406	glyceraldehyde-3-phosphate dehydrogenase
Metabolic	WP4290_118611	P00367	glutamate dehydrogenase 1

reprogramming in colon cancer			
Metabolic reprogramming in colon cancer	WP4290_118611	A2A274	Aconitate hydratase, mitochondrial
Metabolic reprogramming in colon cancer	WP4290_118611	B7Z509	glutaminase
Cancer pathways	WP5434_129459	P08238	heat shock protein 90 alpha family class B member 1
Cancer pathways	WP5434_129459	P14625	heat shock protein 90 beta family member 1
Cancer pathways	WP5434_129459	Q9UKG1	adaptor protein, phosphotyrosine interacting with PH domain and leucine zipper 1
Cancer pathways	WP5434_129459	Q04721	notch receptor 2
Cancer pathways	WP5434_129459	A9UF01	BCR/ABL fusion protein isoform X8
Cancer pathways	WP5434_129459	O15230	laminin subunit alpha 5
Cancer pathways	WP5434_129459	Q03113	G protein subunit alpha 12
Cancer pathways	WP5434_129459	P46109	CRK like proto-oncogene, adaptor protein
Cancer pathways	WP5434_129459	P08754	G protein subunit alpha i3
Cancer pathways	WP5434_129459	P23458	Janus kinase 1
Cancer pathways	WP5434_129459	P01019	angiotensinogen
Cancer pathways	WP5434_129459	Q16204	coiled-coil domain containing 6
Cancer pathways	WP5434_129459	Q9HAV0	G protein subunit beta 4
Cancer pathways	WP5434_129459	P12270	translocated promoter region, nuclear basket protein
Cancer pathways	WP5434_129459	B3KR49	mitogen-activated protein

			kinase
Cancer pathways	WP5434_129459	Q5T8R1	glutathione S-transferase mu 5
Cancer pathways	WP5434_129459	B1AM21	G protein subunit alpha q
Cancer pathways	WP5434_129459	Q08828	adenylate cyclase 1
Cancer pathways	WP5434_129459	P01111	NRAS proto-oncogene, GTPase
Cancer pathways	WP5434_129459	Q13464	Rho associated coiled-coil containing protein kinase 1
Cancer pathways	WP5434_129459	P42345	mechanistic target of rapamycin kinase

Supplementary Table 9. List of EV proteins exclusive in blood associated to every modulated pathway

Biological Process	GO ID	GS	Description
Protein localization to chromosome	GO:0034502	VCP	valosin containing protein
		CCT4	chaperonin containing TCP1 subunit 4
		CCT8	chaperonin containing TCP1 subunit 8
		H4C9	H4 clustered histone 9
		H4C1	H4 clustered histone 1
		H4C4	H4 clustered histone 4
		H4C6	H4 clustered histone 6
		H4C12	H4 clustered histone 12
		H4C11	H4 clustered histone 11
		H4C3	H4 clustered histone 3
		H4C8	H4 clustered histone 8
		H4C2	H4 clustered histone 2
		H4C5	H4 clustered histone 5
		H4C13	H4 clustered histone 13
		H4C14	H4 clustered histone 14
		H4C16	H4 histone 16
		H4C15	H4 clustered histone 15
		CCT3	chaperonin containing TCP1 subunit 3
		H2AC8	H2A clustered histone 8
		H2AC4	H2A clustered histone 4
Protein localization to chromatin	GO:0071168	VCP	valosin containing protein
		H4C9	H4 clustered histone 9
		H4C1	H4 clustered histone 1
		H4C4	H4 clustered histone 4
		H4C6	H4 clustered histone 6
		H4C12	H4 clustered histone 12

		H4C11	H4 clustered histone 11
		H4C3	H4 clustered histone 3
		H4C8	H4 clustered histone 8
		H4C2	H4 clustered histone 2
		H4C5	H4 clustered histone 5
		H4C13	H4 clustered histone 13
		H4C14	H4 clustered histone 14
		H4C16	H4 histone 16
		H4C15	H4 clustered histone 15
		H2AC8	H2A clustered histone 8
		H2AC4	H2A clustered histone 4
		VCP	valosin containing protein
Protein localization to CENP-A containing chromatin	GO:0061644	H4C9	H4 clustered histone 9
		H4C1	H4 clustered histone 1
		H4C4	H4 clustered histone 4
		H4C6	H4 clustered histone 6
		H4C12	H4 clustered histone 12
		H4C11	H4 clustered histone 11
		H4C3	H4 clustered histone 3
		H4C8	H4 clustered histone 8
		H4C2	H4 clustered histone 2
		H4C5	H4 clustered histone 5
		H4C13	H4 clustered histone 13
		H4C14	H4 clustered histone 14
		H4C16	H4 histone 16
		H4C15	H4 clustered histone 15
		H2AC8	H2A clustered histone 8
		H2AC4	H2A clustered histone 4
Protein localization to chromosome, centromeric region	GO:0071459	H4C9	H4 clustered histone 9
		H4C1	H4 clustered histone 1
		H4C4	H4 clustered histone 4
		H4C6	H4 clustered histone 6
		H4C12	H4 clustered histone 12

		H4C11	H4 clustered histone 11
		H4C3	H4 clustered histone 3
		H4C8	H4 clustered histone 8
		H4C2	H4 clustered histone 2
		H4C5	H4 clustered histone 5
		H4C13	H4 clustered histone 13
		H4C14	H4 clustered histone 14
		H4C16	H4 histone 16
		H4C15	H4 clustered histone 15
		H2AC8	H2A clustered histone 8
		H2AC4	H2A clustered histone 4
		PIP4K2A	phosphatidylinositol-5-phosphate 4-kinase type 2 alpha
		H4C9	H4 clustered histone 9
		H4C1	H4 clustered histone 1
		H4C4	H4 clustered histone 4
		H4C6	H4 clustered histone 6
		H4C12	H4 clustered histone 12
Megakaryocyte differentiation	GO:0030219	H4C11	H4 clustered histone 11
		H4C3	H4 clustered histone 3
		H4C8	H4 clustered histone 8
		H4C2	H4 clustered histone 2
		H4C5	H4 clustered histone 5
		H4C13	H4 clustered histone 13
		H4C14	H4 clustered histone 14
		H4C16	H4 histone 16
		H4C15	H4 clustered histone 15
		LTF	lactotransferrin
		H4C9	H4 clustered histone 9
Negative regulation of myeloid cell differentiation	GO:0045638	H4C1	H4 clustered histone 1
		H4C4	H4 clustered histone 4
		H4C6	H4 clustered histone 6

		H4C12	H4 clustered histone 12
		H4C11	H4 clustered histone 11
		H4C3	H4 clustered histone 3
		H4C8	H4 clustered histone 8
		H4C2	H4 clustered histone 2
		H4C5	H4 clustered histone 5
		H4C13	H4 clustered histone 13
		H4C14	H4 clustered histone 14
		H4C16	H4 histone 16
		H4C15	H4 clustered histone 15
		H4C9	H4 clustered histone 9
		H4C1	H4 clustered histone 1
		H4C4	H4 clustered histone 4
		H4C6	H4 clustered histone 6
		H4C12	H4 clustered histone 12
		H4C11	H4 clustered histone 11
Negative regulation of megakaryocyte differentiation	GO:0045653	H4C3	H4 clustered histone 3
		H4C8	H4 clustered histone 8
		H4C2	H4 clustered histone 2
		H4C5	H4 clustered histone 5
		H4C13	H4 clustered histone 13
		H4C14	H4 clustered histone 14
		H4C16	H4 histone 16
		H4C15	H4 clustered histone 15
		H4C9	H4 clustered histone 9
		H4C1	H4 clustered histone 1
		H4C4	H4 clustered histone 4
		H4C6	H4 clustered histone 6
Regulation of megakaryocyte differentiation	GO:0045652	H4C12	H4 clustered histone 12
		H4C11	H4 clustered histone 11
		H4C3	H4 clustered histone 3
		H4C8	H4 clustered histone 8
		H4C2	H4 clustered histone 2

	H4C5	H4 clustered histone 5
	H4C13	H4 clustered histone 13
	H4C14	H4 clustered histone 14
	H4C16	H4 histone 16
	H4C15	H4 clustered histone 15

Supplementary Table 10. List of EV proteins exclusive in plasma associated to every modulated pathway

Biological Process	GO ID	GS	Description
Actin filament organization	GO:0007015	TPM3	tropomyosin 3
		IQGAP1	IQ motif containing GTPase activating protein 1
		STMN1	stathmin 1
		TPM2	tropomyosin 2
		CFL1	cofilin 1
		ARPC3	actin related protein 2/3 complex subunit 3
		CTNNA2	catenin alpha 2
		COTL1	coactosin like F-actin binding protein 1
		SPTAN1	spectrin alpha, non-erythrocytic 1
		TMSB4X	thymosin beta 4 X-linked
		MTPN	myotrophin
		ERMN	ermin
		ACTN1	actinin alpha 1
		MARCKS	myristoylated alanine rich protein kinase C substrate
		PACSIN1	protein kinase C and casein kinase substrate in neurons 1
		WASF1	WASP family member 1
		ACTN2	actinin alpha 2
		SPTBN1	spectrin beta, non-erythrocytic 1
		SDC4	syndecan 4
		NCK1	NCK adaptor protein 1
		CAPZA2	capping actin protein of muscle Z-line subunit alpha 2
		GMFB	glia maturation factor beta

	KCTD13	potassium channel tetramerization domain containing 13
	CDC42EP4	CDC42 effector protein 4
	TAGLN2	transgelin 2
	RHOG	ras homolog family member G
	BIN1	bridging integrator 1
	HSP90B1	heat shock protein 90 beta family member 1
	RGCC	regulator of cell cycle
	PRKCE	protein kinase C epsilon
	ARHGEF2	Rho/Rac guanine nucleotide exchange factor 2
	FSCN1	fascin actin-bundling protein 1
	ACTR2	actin related protein 2
	SORBS1	sorbin and SH3 domain containing 1
	CAPZA1	capping actin protein of muscle Z-line subunit alpha 1
	ARPC5L	actin related protein 2/3 complex subunit 5 like
	DBN1	drebrin 1
	SYNPO	synaptopodin
	PAK1	p21 (RAC1) activated kinase 1
	ADD3	adducin 3
	PYCARD	PYD and CARD domain containing
	ACTR3	actin related protein 3
	CAP2	cyclase associated actin cytoskeleton regulatory protein 2
	PLS3	plastin 3
	EZR	ezrin

	MYO5A	myosin VA
	MYO5B	myosin VB
	PAK3	p21 (RAC1) activated kinase 3
	CAPZB	capping actin protein of muscle Z-line subunit beta
	GBA2	glucosylceramidase beta 2
	SH3KBP1	SH3 domain containing kinase binding protein 1
	MYO6	myosin VI
	PLEC	plectin
	CDC42EP1	CDC42 effector protein 1
	PPFIA1	PTPRF interacting protein alpha 1
	NCK2	NCK adaptor protein 2
	SPTBN2	spectrin beta, non-erythrocytic 2
	CLASP2	cytoplasmic linker associated protein 2
	DLG1	discs large MAGUK scaffold protein 1
	PPP1R9B	protein phosphatase 1 regulatory subunit 9B
	BCAR1	BCAR1 scaffold protein, Cas family member
	CLASP1	cytoplasmic linker associated protein 1
	SH3BP1	SH3 domain binding protein 1
	LIMCH1	LIM and calponin homology domains 1
	DAAM2	dishevelled associated activator of morphogenesis 2
	RASA1	RAS p21 protein activator 1
	MYO1D	myosin ID

		RUFY3	RUN and FYVE domain containing 3
		PXN	paxillin
		PTK2B	protein tyrosine kinase 2 beta
		NF2	NF2, moesin-ezrin-radixin like (MERLIN) tumor suppressor
		TJP1	tight junction protein 1
		ROCK2	Rho associated coiled-coil containing protein kinase 2
		ARHGAP35	Rho GTPase activating protein 35
		DIAPH2	diaphanous related formin 2
		COBL	cordon-bleu WH2 repeat protein
		NCKAP1	NCK associated protein 1
		ROCK1	Rho associated coiled-coil containing protein kinase 1
		TTN	titin
		MTOR	mechanistic target of rapamycin kinase
		SNAP25	synaptosome associated protein 25
		POTEE	POTE ankyrin domain family member E
		TUBB3	tubulin beta 3 class III
		STMN1	stathmin 1
Axonogenesis	GO:0007409	ACTBL2	actin beta like 2
		NEFL	neurofilament light chain
		RAB3A	RAB3A, member RAS oncogene family
		UCHL1	ubiquitin C-terminal hydrolase L1
		CTNNA2	catenin alpha 2

	NCAM1	neural cell adhesion molecule 1
	GDI1	GDP dissociation inhibitor 1
	MAP2	microtubule associated protein 2
	HSP90AB1	heat shock protein 90 alpha family class B member 1
	CNTN1	contactin 1
	GAP43	growth associated protein 43
	STXBP1	syntaxin binding protein 1
	NEFH	neurofilament heavy chain
	PPP3CB	protein phosphatase 3 catalytic subunit beta
	PICALM	phosphatidylinositol binding clathrin assembly protein
	SLC9A6	solute carrier family 9 member A6
	DCLK1	doublecortin like kinase 1
	TRPV2	transient receptor potential cation channel subfamily V member 2
	DPYSL5	dihydropyrimidinase like 5
	RTN4	reticulon 4
	NFASC	neurofascin
	PLXNA4	plexin A4
	APP	amyloid beta precursor protein
	PAK1	p21 (RAC1) activated kinase 1
	NDEL1	nudE neurodevelopment protein 1 like 1
	MAP1B	microtubule associated protein 1B
	PTPRZ1	protein tyrosine phosphatase receptor type Z1
	ATL1	atlastin GTPase 1

	MAP1A	microtubule associated protein 1A
	CHL1	cell adhesion molecule L1 like
	DNM2	dynamin 2
	RAB21	RAB21, member RAS oncogene family
	PAFAH1B1	platelet activating factor acetylhydrolase 1b regulatory subunit 1
	ADCY1	adenylate cyclase 1
	SPP1	secreted phosphoprotein 1
	PAK3	p21 (RAC1) activated kinase 3
	PTPN11	protein tyrosine phosphatase non-receptor type 11
	ALCAM	activated leukocyte cell adhesion molecule
	TBCE	tubulin folding cofactor E
	MAG	myelin associated glycoprotein
	MAPT	microtubule associated protein tau
	NGFR	nerve growth factor receptor
	KIF5C	kinesin family member 5C
	APLP2	amyloid beta precursor like protein 2
	ANK3	ankyrin 3
	NRXN3	neurexin 3
	MAP6	microtubule associated protein 6
	NEO1	neogenin 1
	GSK3B	glycogen synthase kinase 3 beta
	PLXNA2	plexin A2
	KIF5A	kinesin family member 5A

	RGMA	repulsive guidance molecule BMP co-receptor a
	LLGL1	LLGL scribble cell polarity complex component 1
	XK	X-linked Kx blood group antigen, Kell and VPS13A binding protein
	RUFY3	RUN and FYVE domain containing 3
	TNR	tenascin R
	PRKCQ	protein kinase C theta
	SLITRK1	SLIT and NTRK like family member 1
	TRAK1	trafficking kinesin protein 1
	ARHGAP35	Rho GTPase activating protein 35
	ROBO4	roundabout guidance receptor 4
	FGFR2	fibroblast growth factor receptor 2
	EPHB1	EPH receptor B1
	KIF5B	kinesin family member 5B
	SEMA4D	semaphorin 4D
	COBL	cordon-bleu WH2 repeat protein
	PCDHAC2	protocadherin alpha subfamily C, 2
	TOP2B	DNA topoisomerase II beta
	DOCK7	dedicator of cytokinesis 7
	PTPRS	protein tyrosine phosphatase receptor type S
	LAMA2	laminin subunit alpha 2
	USP9X	ubiquitin specific peptidase 9 X-linked

Vesicle-mediated transport in synapse	GO:0099003	NOTCH2	notch receptor 2
		TRIO	trio Rho guanine nucleotide exchange factor
		SNAP25	synaptosome associated protein 25
		RAP1A	RAP1A, member of RAS oncogene family
		STX1B	syntaxin 1B
		CPLX1	complexin 1
		ARHGDIA	Rho GDP dissociation inhibitor alpha
		RAB3A	RAB3A, member RAS oncogene family
		STX1A	syntaxin 1A
		ATP6V1G2	ATPase H+ transporting V1 subunit G2
		ATP6V0A1	ATPase H+ transporting V0 subunit a1
		SNCB	synuclein beta
		HPCA	hippocalcin
		PRRT2	proline rich transmembrane protein 2
		SH3GL2	SH3 domain containing GRB2 like 2, endophilin A1
		SNCG	synuclein gamma
		SYN2	synapsin II
		RAB3B	RAB3B, member RAS oncogene family
		ATP6V1G1	ATPase H+ transporting V1 subunit G1
		PACSIN1	protein kinase C and casein kinase substrate in neurons 1
		ATP6V1E1	ATPase H+ transporting V1

		subunit E1
	ATP6V1A	ATPase H+ transporting V1 subunit A
	STXBP1	syntaxin binding protein 1
	SYN1	synapsin I
	DOC2A	double C2 domain alpha
	PPP3CB	protein phosphatase 3 catalytic subunit beta
	VAC14	VAC14 component of PIKFYVE complex
	SYP	synaptophysin
	AMPH	amphiphysin
	DNAJC5	DnaJ heat shock protein family (Hsp40) member C5
	SH3GL1	SH3 domain containing GRB2 like 1, endophilin A2
	PICALM	phosphatidylinositol binding clathrin assembly protein
	BIN1	bridging integrator 1
	ATP6V0D1	ATPase H+ transporting V0 subunit d1
	SLC17A7	solute carrier family 17 member 7
	DNM1	dynamin 1
	EPS15	epidermal growth factor receptor pathway substrate 15
	SNPH	syntaphilin
	ATP2A2	ATPase sarcoplasmic/endoplasmic reticulum Ca2+ transporting 2
	NAPB	NSF attachment protein beta
	ATP6V1C1	ATPase H+ transporting V1 subunit C1

	AP2M1	adaptor related protein complex 2 subunit mu 1
	DNM3	dynamin 3
	DNM2	dynamin 2
	CANX	calnexin
	SV2B	synaptic vesicle glycoprotein 2B
	AP2B1	adaptor related protein complex 2 subunit beta 1
	VPS35	VPS35 retromer complex component
	AP3D1	adaptor related protein complex 3 subunit delta 1
	SYNJ1	synaptojanin 1
	SH3GL3	SH3 domain containing GRB2 like 3, endophilin A3
	SLC4A8	solute carrier family 4 member 8
	PIP5K1C	phosphatidylinositol-4-phosphate 5-kinase type 1 gamma
	GRIPAP1	GRIP1 associated protein 1
	TOR1A	torsin family 1 member A
	PPFIA3	PTPRF interacting protein alpha 3
	CLCN3	chloride voltage-gated channel 3
	GAK	cyclin G associated kinase
	SCRN1	secernin 1
	CADPS	calcium dependent secretion activator
	CADPS2	calcium dependent secretion activator 2

		SYT9	synaptotagmin 9
		ERC2	ELKS/RAB6-interacting/CAST family member 2
		ITSN2	intersectin 2
		GIT1	GIT ArfGAP 1
		ITSN1	intersectin 1
		SYNJ2	synaptojanin 2
		BSN	bassoon presynaptic cytomatrix protein
		AP2A1	adaptor related protein complex 2 subunit alpha 1
		SYNE1	spectrin repeat containing nuclear envelope protein 1
		UNC13A	unc-13 homolog A
		ROCK1	Rho associated coiled-coil containing protein kinase 1
		PCLO	piccolo presynaptic cytomatrix protein
		SNAP25	synaptosome associated protein 25
		RAP1A	RAP1A, member of RAS oncogene family
		STX1B	syntaxin 1B
		CPLX1	complexin 1
Synaptic vesicle cycle	GO:0099504	ARHGDIA	Rho GDP dissociation inhibitor alpha
		RAB3A	RAB3A, member RAS oncogene family
		STX1A	syntaxin 1A
		ATP6V1G2	ATPase H+ transporting V1 subunit G2
		ATP6V0A1	ATPase H+ transporting V0

		subunit a1
	SNCB	synuclein beta
	PRRT2	proline rich transmembrane protein 2
	SH3GL2	SH3 domain containing GRB2 like 2, endophilin A1
	SNCG	synuclein gamma
	SYN2	synapsin II
	RAB3B	RAB3B, member RAS oncogene family
	ATP6V1G1	ATPase H+ transporting V1 subunit G1
	PACSIN1	protein kinase C and casein kinase substrate in neurons 1
	ATP6V1E1	ATPase H+ transporting V1 subunit E1
	ATP6V1A	ATPase H+ transporting V1 subunit A
	STXBP1	syntaxin binding protein 1
	SYN1	synapsin I
	DOC2A	double C2 domain alpha
	PPP3CB	protein phosphatase 3 catalytic subunit beta
	SYP	synaptophysin
	AMPH	amphiphysin
	DNAJC5	DnaJ heat shock protein family (Hsp40) member C5
	SH3GL1	SH3 domain containing GRB2 like 1, endophilin A2
	PICALM	phosphatidylinositol binding clathrin assembly protein
	BIN1	bridging integrator 1
	ATP6V0D1	ATPase H+ transporting V0

			subunit d1
		SLC17A7	solute carrier family 17 member 7
		DNM1	dynamin 1
		SNPH	syntaphilin
		ATP2A2	ATPase sarcoplasmic/endoplasmic reticulum Ca2+ transporting 2
		NAPB	NSF attachment protein beta
		ATP6V1C1	ATPase H+ transporting V1 subunit C1
		AP2M1	adaptor related protein complex 2 subunit mu 1
		DNM3	dynamin 3
		DNM2	dynamin 2
		CANX	calnexin
		SV2B	synaptic vesicle glycoprotein 2B
		AP2B1	adaptor related protein complex 2 subunit beta 1
		AP3D1	adaptor related protein complex 3 subunit delta 1
		SYNJ1	synaptojanin 1
		SH3GL3	SH3 domain containing GRB2 like 3, endophilin A3
		SLC4A8	solute carrier family 4 member 8
		PIP5K1C	phosphatidylinositol-4-phosphate 5-kinase type 1 gamma
		GRIPAP1	GRIP1 associated protein 1
		TOR1A	torsin family 1 member A
		PPFIA3	PTPRF interacting protein

			alpha 3
		CLCN3	chloride voltage-gated channel 3
		GAK	cyclin G associated kinase
		SCRN1	secernin 1
		CADPS	calcium dependent secretion activator
		CADPS2	calcium dependent secretion activator 2
		SYT9	synaptotagmin 9
		ERC2	ELKS/RAB6-interacting/CAST family member 2
		ITSN2	intersectin 2
		GIT1	GIT ArfGAP 1
		ITSN1	intersectin 1
		SYNJ2	synaptojanin 2
		BSN	bassoon presynaptic cytomatrix protein
		UNC13A	unc-13 homolog A
		ROCK1	Rho associated coiled-coil containing protein kinase 1
		PCLO	piccolo presynaptic cytomatrix protein
		SNAP25	synaptosome associated protein 25
		RAP1A	RAP1A, member of RAS oncogene family
Exocytosis	GO:0006887	VSNL1	visinin like 1
		STX1B	syntaxin 1B
		CPLX1	complexin 1
		VAMP3	vesicle associated membrane protein 3

	RAB3A	RAB3A, member RAS oncogene family
	STX1A	syntaxin 1A
	RAB3C	RAB3C, member RAS oncogene family
	PRRT2	proline rich transmembrane protein 2
	STAM	signal transducing adaptor molecule
	RAB3D	RAB3D, member RAS oncogene family
	CHGA	chromogranin A
	GNAI2	G protein subunit alpha i2
	SYN2	synapsin II
	RAB15	RAB15, member RAS oncogene family
	RAB3B	RAB3B, member RAS oncogene family
	RPH3AL	rabphilin 3A like (without C2 domains)
	SDC4	syndecan 4
	STXBP1	syntaxin binding protein 1
	SYN1	synapsin I
	DOC2A	double C2 domain alpha
	PPP3CB	protein phosphatase 3 catalytic subunit beta
	PTGDS	prostaglandin D2 synthase
	SYP	synaptophysin
	DNAJC5	DnaJ heat shock protein family (Hsp40) member C5
	RAB33B	RAB33B, member RAS oncogene family
	SNPH	syntaphilin

	RABGEF1	RAB guanine nucleotide exchange factor 1
	ATP2A2	ATPase sarcoplasmic/endoplasmic reticulum Ca2+ transporting 2
	NAPB	NSF attachment protein beta
	PAK1	p21 (RAC1) activated kinase 1
	RAB21	RAB21, member RAS oncogene family
	SV2B	synaptic vesicle glycoprotein 2B
	LYN	LYN proto-oncogene, Src family tyrosine kinase
	SDC1	syndecan 1
	SYNJ1	synaptojanin 1
	IL1RAPL1	interleukin 1 receptor accessory protein like 1
	RAB11FIP5	RAB11 family interacting protein 5
	SLC4A8	solute carrier family 4 member 8
	RAB11FIP1	RAB11 family interacting protein 1
	PIP5K1C	phosphatidylinositol-4-phosphate 5-kinase type 1 gamma
	ATP9A	ATPase phospholipid transporting 9A (putative)
	PPFIA3	PTPRF interacting protein alpha 3
	EXOC6B	exocyst complex component 6B
	CLASP2	cytoplasmic linker associated

			protein 2
		SCRN1	secernin 1
		LLGL1	LLGL scribble cell polarity complex component 1
		CADPS	calcium dependent secretion activator
		CADPS2	calcium dependent secretion activator 2
		CLASP1	cytoplasmic linker associated protein 1
		MIA3	MIA SH3 domain ER export factor 3
		SYT9	synaptotagmin 9
		ERC2	ELKS/RAB6-interacting/CAST family member 2
		GIT1	GIT ArfGAP 1
		ITSN1	intersectin 1
		EXOC8	exocyst complex component 8
		EXOC4	exocyst complex component 4
		LLGL2	LLGL scribble cell polarity complex component 2
		EXOC1	exocyst complex component 1
		ARFGEF2	ADP ribosylation factor guanine nucleotide exchange factor 2
		UNC13A	unc-13 homolog A
		PCLO	piccolo presynaptic cytomatrix protein
Protein polymerization	GO:0051258	TUBB4A	tubulin beta 4A class IVa
		STMN1	stathmin 1
		TUBB1	tubulin beta 1 class VI
		NEFL	neurofilament light chain

	ARPC3	actin related protein 2/3 complex subunit 3
	TPPP	tubulin polymerization promoting protein
	COTL1	coactosin like F-actin binding protein 1
	SPTAN1	spectrin alpha, non-erythrocytic 1
	TMSB4X	thymosin beta 4 X-linked
	TPPP3	tubulin polymerization promoting protein family member 3
	MTPN	myotrophin
	MAP2	microtubule associated protein 2
	WASF1	WASP family member 1
	SPTBN1	spectrin beta, non-erythrocytic 1
	NDE1	nudE neurodevelopment protein 1
	NCK1	NCK adaptor protein 1
	CAPZA2	capping actin protein of muscle Z-line subunit alpha 2
	CDC42EP4	CDC42 effector protein 4
	BIN1	bridging integrator 1
	PRKCE	protein kinase C epsilon
	UBE2K	ubiquitin conjugating enzyme E2 K
	MAPRE1	microtubule associated protein RP/EB family member 1
	CAPZA1	capping actin protein of muscle Z-line subunit alpha 1
	ARPC5L	actin related protein 2/3

		complex subunit 5 like
	PAK1	p21 (RAC1) activated kinase 1
	NDEL1	nudE neurodevelopment protein 1 like 1
	RPS3	ribosomal protein S3
	ADD3	adducin 3
	PYCARD	PYD and CARD domain containing
	MAP1B	microtubule associated protein 1B
	CHMP4B	charged multivesicular body protein 4B
	PAK3	p21 (RAC1) activated kinase 3
	CAPZB	capping actin protein of muscle Z-line subunit beta
	VDAC2	voltage dependent anion channel 2
	GBA2	glucosylceramidase beta 2
	TTBK1	tau tubulin kinase 1
	PSRC1	proline and serine rich coiled-coil 1
	MAPT	microtubule associated protein tau
	CKAP5	cytoskeleton associated protein 5
	CDC42EP1	CDC42 effector protein 1
	NCK2	NCK adaptor protein 2
	SPTBN2	spectrin beta, non-erythrocytic 2
	CLASP2	cytoplasmic linker associated protein 2
	DLG1	discs large MAGUK scaffold protein 1

		CLASP1	cytoplasmic linker associated protein 1
		DAAM2	dishevelled associated activator of morphogenesis 2
		RASA1	RAS p21 protein activator 1
		PTK2B	protein tyrosine kinase 2 beta
		NUMA1	nuclear mitotic apparatus protein 1
		GIT1	GIT ArfGAP 1
		TRIM32	tripartite motif containing 32
		CLIP1	CAP-Gly domain containing linker protein 1
		DIAPH2	diaphanous related formin 2
		COBL	cordon-bleu WH2 repeat protein
		MAP4	microtubule associated protein 4
		NCKAP1	NCK associated protein 1
		MTOR	mechanistic target of rapamycin kinase
		RAB3A	RAB3A, member RAS oncogene family
		STAM	signal transducing adaptor molecule
		SYN2	synapsin II
Vesicle localization	GO:0051648	MAP2	microtubule associated protein 2
		DCTN2	dynactin subunit 2
		SDC4	syndecan 4
		NDE1	nudE neurodevelopment protein 1
		SYN1	synapsin I
		PICALM	phosphatidylinositol binding

		clathrin assembly protein
	DNM1	dynamin 1
	ACTN4	actinin alpha 4
	TPGS1	tubulin polyglutamylase complex subunit 1
	GBF1	golgi brefeldin A resistant guanine nucleotide exchange factor 1
	NDEL1	nudE neurodevelopment protein 1 like 1
	USO1	USO1 vesicle transport factor
	DNM2	dynamin 2
	PAFAH1B1	platelet activating factor acetylhydrolase 1b regulatory subunit 1
	ARFGAP2	ADP ribosylation factor GTPase activating protein 2
	STARD3NL	STARD3 N-terminal like
	MYO5A	myosin VA
	AP3D1	adaptor related protein complex 3 subunit delta 1
	MYO5B	myosin VB
	SDC1	syndecan 1
	SYNJ1	synaptojanin 1
	DYNC1I1	dynein cytoplasmic 1 intermediate chain 1
	VPS33B	VPS33B late endosome and lysosome associated
	MYO6	myosin VI
	ATP9A	ATPase phospholipid transporting 9A (putative)
	TOR1A	torsin family 1 member A
	EXOC6B	exocyst complex component

			6B
		KIF5C	kinesin family member 5C
		CLASP2	cytoplasmic linker associated protein 2
		KIF5A	kinesin family member 5A
		CLASP1	cytoplasmic linker associated protein 1
		MYO1D	myosin ID
		SYBU	syntabulin
		TRAK1	trafficking kinesin protein 1
		TRAPPC9	trafficking protein particle complex subunit 9
		EXOC8	exocyst complex component 8
		BSN	bassoon presynaptic cytomatrix protein
		EXOC4	exocyst complex component 4
		KIF5B	kinesin family member 5B
		EXOC1	exocyst complex component 1
		WDR11	WD repeat domain 11
		PCLO	piccolo presynaptic cytomatrix protein
Synaptic vesicle recycling	GO:0036465	STX1B	syntaxin 1B
		RAB3A	RAB3A, member RAS oncogene family
		STX1A	syntaxin 1A
		SNCB	synuclein beta
		SH3GL2	SH3 domain containing GRB2 like 2, endophilin A1
		SNCG	synuclein gamma
		PACSIN1	protein kinase C and casein kinase substrate in neurons 1
		PPP3CB	protein phosphatase 3 catalytic subunit beta

	SYP	synaptophysin
	AMPH	amphiphysin
	SH3GL1	SH3 domain containing GRB2 like 1, endophilin A2
	PICALM	phosphatidylinositol binding clathrin assembly protein
	BIN1	bridging integrator 1
	SLC17A7	solute carrier family 17 member 7
	DNM1	dynamin 1
	AP2M1	adaptor related protein complex 2 subunit mu 1
	DNM3	dynamin 3
	DNM2	dynamin 2
	CANX	calnexin
	AP2B1	adaptor related protein complex 2 subunit beta 1
	AP3D1	adaptor related protein complex 3 subunit delta 1
	SYNJ1	synaptojanin 1
	SH3GL3	SH3 domain containing GRB2 like 3, endophilin A3
	PIP5K1C	phosphatidylinositol-4-phosphate 5-kinase type 1 gamma
	GRIPAP1	GRIP1 associated protein 1
	TOR1A	torsin family 1 member A
	GAK	cyclin G associated kinase
	ITSN2	intersectin 2
	GIT1	GIT ArfGAP 1
	ITSN1	intersectin 1
	SYNJ2	synaptojanin 2
	ROCK1	Rho associated coiled-coil

			containing protein kinase 1
		STX1A	syntaxin 1A
		SNCB	synuclein beta
		SH3GL2	SH3 domain containing GRB2 like 2, endophilin A1
		SNCG	synuclein gamma
		PACSIN1	protein kinase C and casein kinase substrate in neurons 1
		PPP3CB	protein phosphatase 3 catalytic subunit beta
		SYP	synaptophysin
		AMPH	amphiphysin
		SH3GL1	SH3 domain containing GRB2 like 1, endophilin A2
		PICALM	phosphatidylinositol binding clathrin assembly protein
Synaptic vesicle endocytosis	GO:0048488	BIN1	bridging integrator 1
		SLC17A7	solute carrier family 17 member 7
		DNM1	dynamin 1
		AP2M1	adaptor related protein complex 2 subunit mu 1
		DNM3	dynamin 3
		DNM2	dynamin 2
		CANX	calnexin
		AP2B1	adaptor related protein complex 2 subunit beta 1
		AP3D1	adaptor related protein complex 3 subunit delta 1
		SYNJ1	synaptojanin 1
		SH3GL3	SH3 domain containing GRB2 like 3, endophilin A3
		PIP5K1C	phosphatidylinositol-4-

			phosphate 5-kinase type 1 gamma
		TOR1A	torsin family 1 member A
		GAK	cyclin G associated kinase
		ITSN2	intersectin 2
		ITSN1	intersectin 1
		SYNJ2	synaptojanin 2
		ROCK1	Rho associated coiled-coil containing protein kinase 1
		STX1A	syntaxin 1A
		SNCB	synuclein beta
		SH3GL2	SH3 domain containing GRB2 like 2, endophilin A1
		SNCG	synuclein gamma
		PACSIN1	protein kinase C and casein kinase substrate in neurons 1
		PPP3CB	protein phosphatase 3 catalytic subunit beta
		SYP	synaptophysin
		AMPH	amphiphysin
Presynaptic endocytosis	GO:0140238	SH3GL1	SH3 domain containing GRB2 like 1, endophilin A2
		PICALM	phosphatidylinositol binding clathrin assembly protein
		BIN1	bridging integrator 1
		SLC17A7	solute carrier family 17 member 7
		DNM1	dynamin 1
		AP2M1	adaptor related protein complex 2 subunit mu 1
		DNM3	dynamin 3
		DNM2	dynamin 2
		CANX	calnexin

	AP2B1	adaptor related protein complex 2 subunit beta 1
	AP3D1	adaptor related protein complex 3 subunit delta 1
	SYNJ1	synaptojanin 1
	SH3GL3	SH3 domain containing GRB2 like 3, endophilin A3
	PIP5K1C	phosphatidylinositol-4-phosphate 5-kinase type 1 gamma
	TOR1A	torsin family 1 member A
	GAK	cyclin G associated kinase
	ITSN2	intersectin 2
	ITSN1	intersectin 1
	SYNJ2	synaptojanin 2
	ROCK1	Rho associated coiled-coil containing protein kinase 1

Supplementary Table 11. Proteins classified as nVEP according to the MISEV guidelines, including major components of non-EV co-isolated structures as well as transmembrane, lipid-bound, and soluble proteins associated with intracellular compartments other than the plasma membrane or endosomes, identified in the proteomic analysis of PROSPR-enriched blood-derived EV preparations. For each protein, the accession number, GS, protein description, and spectral count (mean ± SD) are reported.

**Accession**	**GS**	**Description**	**Average spectral count ± SD**
P02768	ALB	Albumin	746,667 ± 424,975
A0A024R3E3	APOA1	Apolipoprotein A-I	46,333 ± 28,335
P00338	LDHA	L-lactate dehydrogenase A chain	10,667 ± 7,717
A3KPE2	APOC3	Apolipoprotein C-III	8,000 ± 5,354
V9GYG9	APOA2	Apolipoprotein A-II	7,333 ± 3,399
P05090	APOD	Apolipoprotein D	7,000 ± 4,899
A0A024R0T9	APOC2	Apolipoprotein C-II	5,333 ± 3,859
P62805	H4C9	Histone H4	4,667 ± 0,471
P02649	APOE	Apolipoprotein E	4,667 ± 4,643
P06727	APOA4	Apolipoprotein A-IV	4,000 ± 3,266
B2RA03	KRT18	Keratin 18	3,667 ± 0,943
Q08AJ9	H2AC8	Histone H2A	3,667 ± 1,247
A0A7P0TB36	HSPA5	Endoplasmic reticulum chaperone BIP	2,000 ± 1,414
A4FVC0	AGO2	Protein argonaute 2	1,667 ± 1,700
K7ERI9	APOC1	Apolipoprotein C-I	1,333 ± 1,886
P08519	LPA	Apolipoprotein(a)	1,000 ± 1,414
A0A804HLF4	ACTN1	Actinin alpha 1	0,333 ± 0,471
A0A1U9X793	APOM	Apolipoprotein M	0,333 ± 0,471

nVEP: Non-vesicular extracellular proteins; MISEV: Minimal Information for Studies of Extracellular Vesicles; PROSPR: PRotein Organic Solvent PRecipitation; EV: extracellular vesicle; GS: gene symbol; SD: standard deviation.
